# Amino Acid Metabolism-Regulated Nanomedicine for Enhanced Tumor Immunotherapy through Synergistic Regulation of Immune Microenvironment

**DOI:** 10.34133/bmr.0048

**Published:** 2024-07-04

**Authors:** Xiuying Duan, Yilei Zhao, Houyang Hu, Xuechun Wang, Jie Yan, Songyan Li, Yueying Zhang, Jianwei Jiao, Guiqiang Zhang

**Affiliations:** ^1^ Medical Science and Technology Innovation Center, Shandong First Medical University & Shandong Academy of Medical Sciences, Jinan, Shandong 250117, China.; ^2^ School of Life Sciences, Shandong First Medical University & Shandong Academy of Medical Sciences, Jinan, Shandong 250117, China.; ^3^ School of Clinical and Basic Medical Sciences, Shandong First Medical University & Shandong Academy of Medical Sciences, Jinan, Shandong 250117, China.; ^4^ State Key Laboratory of Stem Cell and Reproductive Biology, Institute of Zoology, Chinese Academy of Sciences, Beijing 100101, China.

## Abstract

The reprogramming of tumor metabolism presents a substantial challenge for effective immunotherapy, playing a crucial role in developing an immunosuppressive microenvironment. In particular, the degradation of the amino acid L-tryptophan (Trp) to kynurenine (Kyn) by indoleamine-pyrrole 2,3-dioxygenase 1 (IDO1) is one of the most clinically validated pathways for immune suppression. Thus, regulating the Trp/Kyn metabolism by IDO1 inhibition represents a promising strategy for enhancing immunotherapy. Herein, metabolism-regulated nanoparticles are prepared through metal coordination-driven assembly of an IDO1 inhibitor (NLG919) and a stimulator of interferon genes (STING) agonist (MSA-2) for enhanced immunotherapy. After intravenous administration, the assembled nanoparticles could efficiently accumulate in tumors, enhancing the bioavailability of NLG919 and down-regulating the metabolism of Trp to Kyn to remodel the immunosuppressive tumor microenvironment. Meanwhile, the released MSA-2 evoked potent STING pathway activation in tumors, triggering an effective immune response. The antitumor immunity induced by nanoparticles significantly inhibited the development of primary and metastatic tumors, as well as B16 melanoma. Overall, this study provided a novel paradigm for enhancing tumor immunotherapy through synergistic amino acid metabolism and STING pathway activation.

## Introduction

Immunotherapy, which stimulates the body,s immune system to attack tumor cells and induces long-term immunological memory, has shown significant promise in cancer therapy [1,2]. However, the tumor-created immunosuppressive microenvironment severely hampers its therapeutic outcome [3,4]. Tumor immunosuppression is highly correlated with metabolic alterations and metabolites accumulated in the tumor microenvironment (TME), including glucose, lactate, amino acids, and adenosine [5–8]. In particular, the degradation of the amino acid L-tryptophan (Trp) to kynurenine (Kyn) by indoleamine-pyrrole 2,3-dioxygenase 1 (IDO1) is one of the most clinically validated pathways for immune suppression in tumors [9,10]. Tryptophan is an essential amino acid for T-cell activation and proliferation, and its deficiency can inhibit T cells by down-regulating the mTORC1 complex and up-regulating general control nonderepressible 2 (GCN2) [11,12]. Meanwhile, the accumulation of Kyn within TME promotes the activity of regulatory T cells (Tregs) and jeopardizes the function of CD8^+^ T cells and natural killer (NK) cells [13]. Thus, regulating the Trp/Kyn metabolism by IDO1 inhibition represents a promising strategy for reprograming the immunosuppressive TME.

Recently, several small-molecule IDO1 inhibitors, such as NLG919, 1-methyl-L-tryptophan, and epacadostat, have been developed to block the IDO1 pathway [14,15]. However, their low bioavailability has hindered their biological application due to poor water solubility and a short half-life [16,17]. Furthermore, the systemic administration of IDO1 inhibitors may induce some off-target side effects. Various nanoparticles (NPs), including liposomes, inorganic NPs, and polymer NPs, have been investigated to enhance the bioavailability and therapeutic efficacy of cargoes, highlighting the potential of NP-based delivery systems for IDO1 inhibitors [18–20]. However, tumor immunotherapy based solely on IDO1 inhibition may produce limited therapeutic effects in practice due to the scarce infiltration of CD8^+^ T cells in tumors [21]. Therefore, combinatorial regimens employing IDO inhibitors and other therapeutic strategies that recruit more CD8^+^ T cells are urgently needed.

Activating the stimulator of interferon genes (STING) signaling pathway represents an emerging strategy for enhancing antitumor immunity [22–24]. The STING pathway can up-regulate type I interferons (e.g., IFN-β) and pro-inflammatory cytokines to trigger specific immune responses via activating and infiltrating CD8^+^ T and NK cells [25–27]. Thus, a combination of IDO1 inhibition and STING activation could integrate the advantages of regulating Trp/Kyn metabolism and the intratumoral infiltration of T cells, which might help remodel the immunosuppressive TME. As one of the most commonly used STING agonists, cyclic dinucleotides (CDNs) can only be administered through the intratumoral route due to metabolic instability and poor cellular permeability [28]. Unfortunately, the efficacy of CDNs alone or combined with immune checkpoint blockade (ICB) in phase I clinical trials was almost zero [29,30]. Although small-molecule STING agonists with good stability have been recently investigated for systemic administration, their poor bioavailability and off-target inflammatory responses still pose significant challenges [31,32]. For more effective immunotherapy, developing a new nanomedicine capable of efficiently co-delivering STING agonists and IDO1 inhibitors to tumor tissues is essential.

**Fig. 1.**
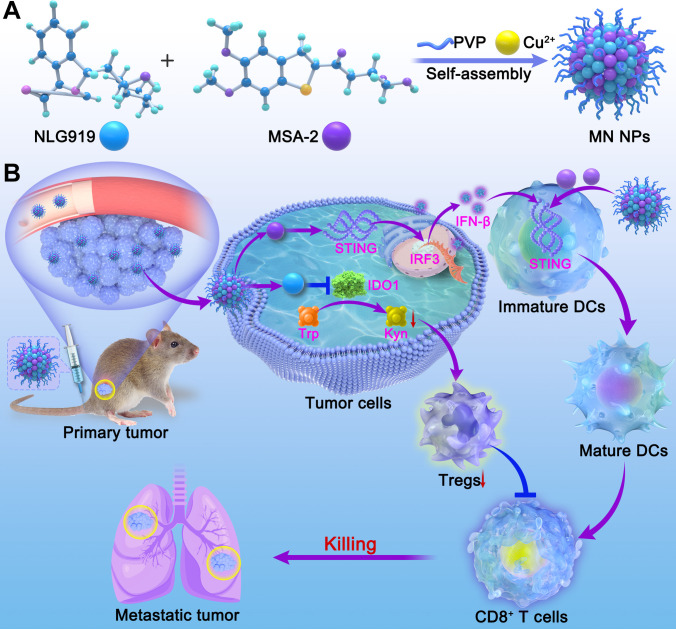
Schematic illustration of MN NPs for enhanced tumor immunotherapy. (A) Preparation of MN NPs via the assembly of NLG919, MSA-2, and Cu^2+^. (B) Accumulation of NPs in tumors and the induced antitumor immunity (e.g., DC maturation, T-cell activation, and reduction of Tregs) resulting in primary and metastatic tumor inhibition.

Coordination-driven assembly presents a compelling approach for fabricating delivery systems for small-molecule drugs, nucleic acids, and proteins due to its convenient preparation conditions, robust stability of the coordination bonds, and high drug-loading capacity [33–36]. In addition, potential carrier-induced side effects (e.g., low biocompatibility and immunogenicity) are minimized using therapeutic molecules as the coordinating ligands [37,38]. Herein, metabolism-regulated NPs (termed MN NPs) were fabricated by the self-assembly of a small-molecule IDO1 inhibitor (i.e., NLG919), STING agonist (i.e., MSA-2), and copper ions (Cu^2+^) based on metal coordination and noncovalent interactions for immunotherapy. The obtained NPs could accumulate in tumors to reverse the immunosuppressive TME and elicit antitumor immunity, including dendritic cell (DC) maturation, Tregs reduction, and effector T-cell infiltration, resulting in remarkable antitumor efficacy (Fig. 1).

## Materials and Methods

### Materials

NLG919, MSA-2, ICG, polyvinylpyrrolidone (PVP), and methylthiazolyldiphenyl-tetrazolium bromide (MTT) were obtained from MedChemExpress (China). Enzyme-linked immunosorbent assay (ELISA) kits for cytokine detection and fluorochrome-conjugated anti-mouse antibodies for flow cytometry were procured from BioLegend (USA) and eBioscience (USA), respectively. Other chemicals were obtained from Sinopharm Co. Ltd. (China) unless noted.

### Preparation of NPs

Stock solutions of NLG919 in ethanol (11.3 mg ml^−1^), MSA-2 in dimethyl sulfoxide (DMSO, 8 mg ml^−1^), PVP in water (5 mg ml^−1^), and CuCl_2_·4H_2_O in water (40 mM) were freshly prepared. Subsequently, 100 μl of NLG919, 50 μl of MSA-2, and 35 μl of Cu^2+^ solutions were mixed, followed by the addition of 815 μl of water and 100 μl of PVP under vigorous stirring. Following centrifugation and washing with water, MN NPs were obtained and stored for future use.

### Characterization

A Malvern Zetasizer (Nano ZS90, UK) was used to investigate the hydrodynamic size and zeta potential of NPs. The morphology of NPs was examined using a transmission electron microscope (JEOL JEM-1400, Japan) and a scanning electron microscope (Zeiss G300, Germany). UV–Vis absorbance spectra were obtained using a Shimadzu UV-2600 spectrophotometer (Japan). The amounts of NLG919 and MSA-2 in NPs were measured by UV–Vis absorbance spectra.

### Cell cytotoxicity

The cytotoxicity of the obtained NPs against 4T1 cells was measured via an MTT assay as described previously [39].

### Cellular uptake

Fluorescently labeled MN NPs (ICG-NPs) were prepared by incorporating ICG during the assembly of NPs. 4T1 cells (5 × 10^4^ cells per well) were incubated in 24-well plates and treated with ICG and ICG-NPs at the same concentration of ICG for 24 h. The cells were examined using flow cytometry (NovoCyte 2060R, ACEA, USA) and confocal laser scanning microscopy (CLSM) (TCP SP8 STED 3X, Leica, Germany).

### IDO pathway inhibition assay

4T1 cells (10^5^ cells per well) were incubated in 12-well plates and treated with 100 ng ml^−1^ of IFN-γ for 24 h. After further treatment with PBS, NLG919, MSA-2, and MN NPs at equivalent concentrations for 24 h, the cells were lysed. The supernatant was collected, and its Kyn content was measured using an ELISA kit. The precipitated proteins were loaded onto sodium dodecyl sulfate polyacrylamide gel electrophoresis (SDS-PAGE) gels (15 μg per lane) and transferred to polyvinylidene fluoride (PVDF) membranes. The membranes were incubated with primary antibodies (anti-IDO1 [654002, BioLegend] and anti-*β*-actin [AF2811, Beyotime, China]) at 4°C for 12 h. After incubation with HRP-conjugated goat anti-rabbit IgG, the blot signals were visualized using an ECL reagent.

### STING pathway activation assay

Following incubation with MN NPs for 24 h, the supernatant of DC2.4 cells was collected for an ELISA assay to examine IFN-β expression. DC2.4 (2 × 10^5^ cells) and 4T1 cells (10^5^ cells) were lysed and loaded onto SDS-PAGE gels (15 μg per lane), followed by transfer to PVDF membranes. The membranes were incubated with primary antibodies (anti-pTBK1 (3504, Cell Signaling Technology, USA), anti-pSTING (50494, Cell Signaling Technology), anti-pIRF3 (4302, Cell Signaling Technology), and anti-*β*-actin) at 4°C for 12 h. After incubation with HRP-conjugated goat anti-rabbit IgG, the blot signals were visualized using an ECL reagent (Cell Signaling Technology, USA).

### DC maturation in vitro

BMDCs were obtained as described previously [40]. BMDCs were stained with antibodies (anti-CD11c, anti-CD86, and anti-CD80) after a 24-h incubation with various formulations and analyzed using flow cytometry.

### Animal study

Female BALB/c mice were sourced from Vital River Laboratory Animal Technology Co. Ltd (China). To establish the tumor model, 4T1 cells (1 × 10^6^) in 100 μl of PBS were subcutaneously injected into the flanks of each mouse. The tumor volume (*V*) was calculated using the following equation: *V =* 1/2 *×* length *×* width^2^.

### Biodistribution of NPs

When the tumor volume was 200 mm^3^, the fluorescently labeled NPs were intravenously injected at an ICG dose of 2 mg kg^−1^. Fluorescence images were captured in vivo using a fluorescence imaging system (PerkinElmer; IVIS Spectrum, USA). Major organs were collected 24 h post-injection to examine the distribution of NPs.

### Antitumor effect and biosafety assay

Upon reaching a tumor volume of 100 mm^3^, the mice were randomly assigned to groups and administered intravenously with PBS, NLG919, MSA-2, and MN NPs at equivalent concentrations of NLG919 (5 mg kg^−1^) and MSA-2 (2.4 mg kg^−1^). The treatment was repeated every 2 days for a total of 3 times. The body weight and tumor volume of the mice were monitored every 2 days. On day 18 post-treatment, the serum was collected and analyzed to assess the biosafety.

### Antitumor immunity in vivo

The inguinal LNs from mice were processed to yield single lymphocytes for DC maturation analysis. Following staining with fluorescently labeled antibodies (anti-CD11c, anti-CD80, and anti-CD86), the DC population was quantified using flow cytometry. Spleen lymphocytes were obtained and stained with the fluorescently labeled antibodies (anti-CD3, anti-CD4, anti-CD8, anti-CD44, and anti-CD62L) to assess immune activation and immune memory. Subsequently, the tumors were excised, homogenized, and digested to obtain single cells. After staining with fluorescently labeled antibodies, the infiltrations of CD8^+^ T cells (CD3^+^CD8^+^), CD4^+^ T cells (CD3^+^CD4^+^), and Tregs (CD3^+^CD4^+^Foxp3^+^) in tumors were quantified using flow cytometry.

### Transcriptomic analysis

Each mouse received a subcutaneous injection of 4T1 cells (1 × 10^6^) in 100 μl of PBS into the right flank. Upon reaching a tumor volume of 100 mm^3^, the mice were intravenously administered MN NPs at a dose equivalent to 5 mg kg^−1^ of NLG919 and 2.4 mg kg^−1^ of MSA-2. The treatment was repeated every 2 days for a total of 3 times. After 24 h of injection of the last dose, the tumors were collected for transcriptomic analysis.

### Lung metastasis inhibition

When the primary tumor volume in the right flank reached 100 mm^3^, the mice were intravenously injected with 1 × 10^6^ 4T1 cells. After 24 h, the mice received intravenous injections of PBS, aPD-L1, MN NPs, and MN NPs + aPD-L1 at an NLG919 dose of 5 mg kg^−1^ and an aPD-L1 dose of 5 mg kg^−1^. The treatment was repeated every 2 days for a total of three cycles. On day 10 of the final treatment, all the lungs were harvested, photographed, and stained with H&E.

### Antitumor efficacy in the B16 tumor model

To establish the tumor model, B16 cells (1 × 10^6^) in 100 μl of PBS were subcutaneously injected into the flanks of each mouse. Once the tumor reached a volume of 100 mm^3^, the mice were intravenously injected with PBS, NLG919, MSA-2, and MN NPs at equivalent concentrations of NLG919 (5 mg kg^−1^) and MSA-2 (2.4 mg kg^−1^). The treatment was repeated every 2 days for a total of 3 times. The body weight, tumor volume, and antitumor immunity were assessed following the description provided for the 4T1 tumor model.

### Statistical analysis

Results are expressed as the mean ± SD. For the comparison between 2 groups, a 2-tailed Student’s *t* test was conducted, while one-way ANOVA was used for multiple-group analysis.

## Results

### Synthesis and characterization of MN NPs

**Fig. 2.**
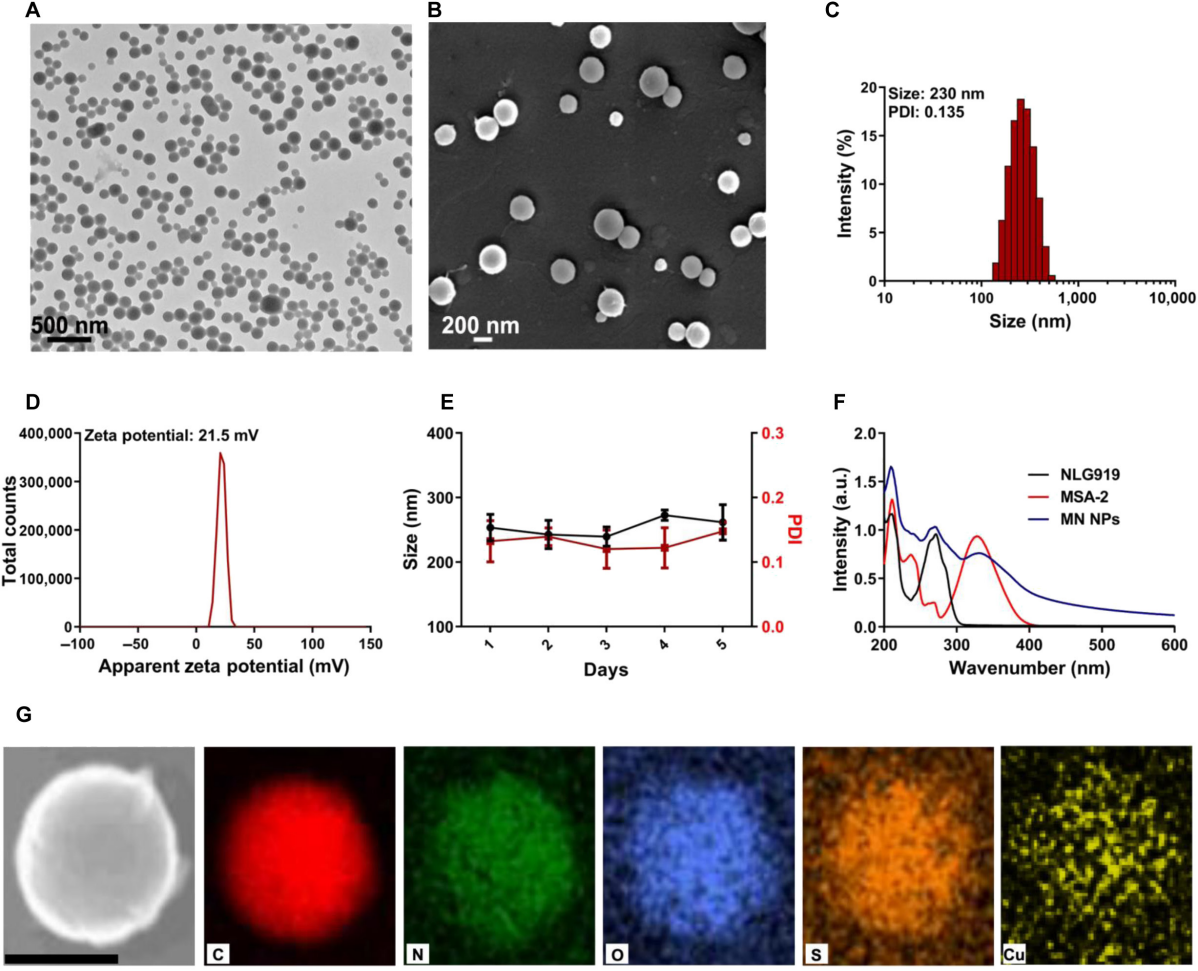
Characterization of MN NPs. (A) TEM, (B) SEM images, (C) size distribution, (D) zeta potential, (E) colloidal stability, (F) UV–Vis absorbance spectra, and (G) the corresponding elemental mapping images of MN NPs. Scale bars are 200 nm.

Increasing evidence suggests that Cu^2+^ can chelate with hydrophobic NLG919, enhancing its stability and therapeutic efficacy [41,42]. In addition, since the carboxyl group of MSA-2 can participate in metal coordination, Cu^2+^ was chosen for NP assembly. As shown in Fig. 1A, MN NPs were prepared via the one-pot incubation of NLG919, MSA-2, and Cu^2+^ in an aqueous solution under consistent shaking. Also, a biocompatible PVP was introduced to enhance the stability of NPs. Transmission electron microscopy (TEM) and scanning electron microscopy (SEM) were conducted to investigate the morphology of MN NPs. The results revealed that the NPs were uniformly dispersed and had spherical morphology with a diameter of about 220 nm (Fig. 2A and B). The dynamic light scattering (DLS) analysis revealed an approximate hydrodynamic diameter of 230 nm for the MN NPs (Fig. 2C), aligning with observations from TEM and SEM. Additionally, the morphology, size, and polydispersity index (PDI) of the NPs could be altered by adjusting the Cu^2+^ amount (Fig. S1). The zeta potential of the MN NPs was about 21.5 mV (Fig. 2D). The hydrodynamic diameter and PDI of the obtained NPs suggested no obvious variations within 5 days, demonstrating the colloidal stability of MN NPs (Fig. 2E). After incubation with Dulbecco,s modified Eagle medium (DMEM) containing 10% fetal bovine serum, the zeta potential values of MN NPs reversed from positive to negative and their size slightly increased (Fig. S2). Similarly, MN NPs could maintain good colloidal dispersity and colloidal stability in DMEM (Fig. S3). Then, the ultraviolet-visible (UV–Vis) absorbance spectra of the MN NPs were detected. As indicated in Fig. 2F, the characteristic absorption peaks of NLG919 (271 nm) and MSA-2 (327 nm) demonstrated their presence in MN NPs. UV–Vis absorbance spectra were also used to quantify the composition percentage of NLG919 and MSA-2 in the NPs, which was about 64.9% and 31.1%, respectively (Figs. S4 and S5). The elemental mapping images showed that Cu was uniformly distributed in the MN NPs (Fig. 2G), and this presence was further confirmed through energy-dispersive x-ray spectroscopy analysis (Fig. S6). Subsequently, the drug release behavior of MN NPs in the absence or presence of glutathione (GSH) at varying pH values was detected using high-pressure liquid chromatography (HPLC). As depicted in Fig. S7, only 25% of the drug was released from MN NPs within 24 h under neutral and acidic pH environment. Conversely, in the presence of GSH, a burst-release phenomenon occurred within the first 2 h, followed by a gradual release until reaching a plateau. This release pattern was ascribed to the competition coordination between GSH and Cu^2+^.

### Cytotoxicity and cellular uptake

**Fig. 3.**
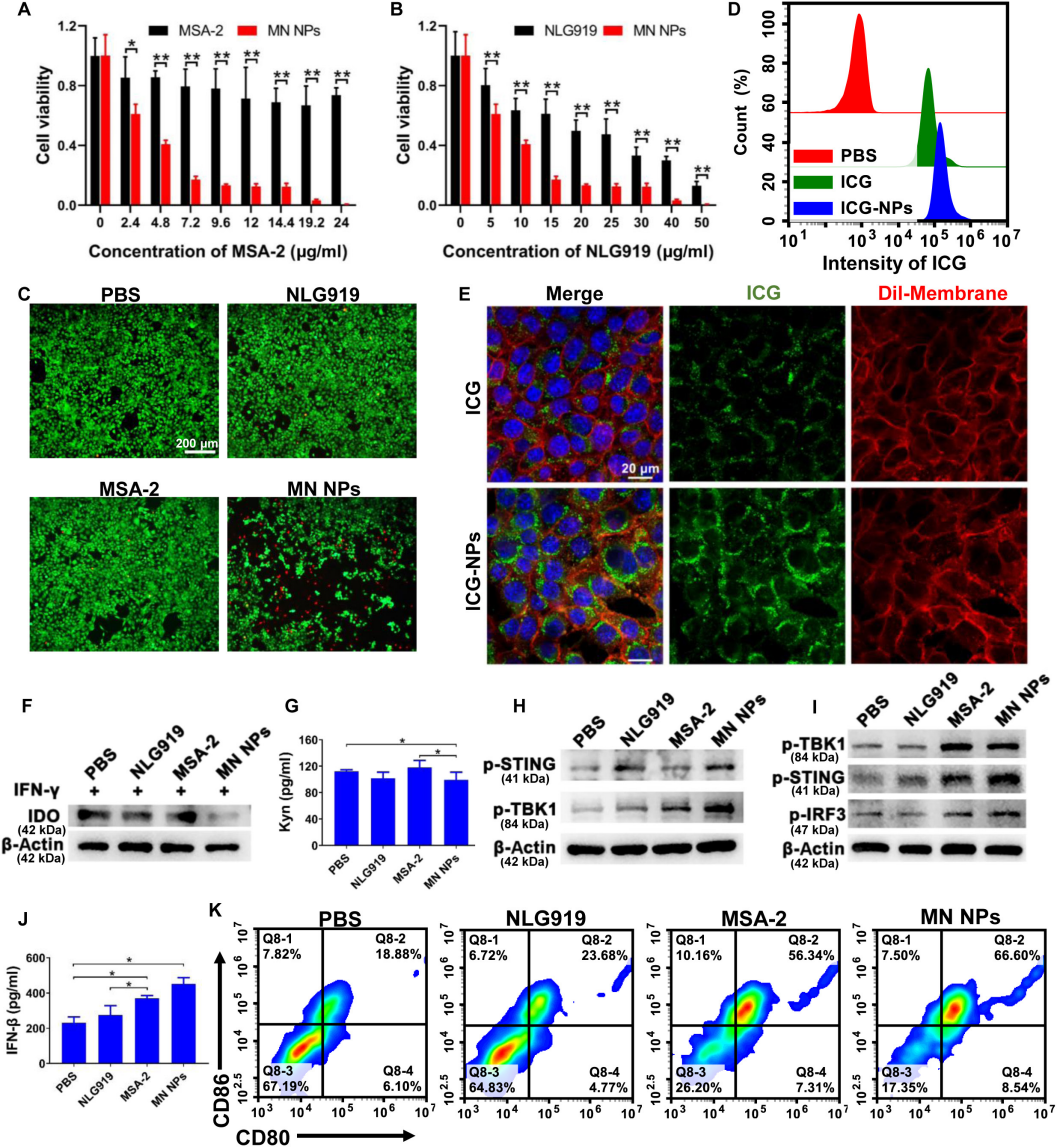
Cytotoxicity, IDO inhibition, and STING activation of NPs. (A and B) Cytotoxicity of MSA-2, NLG919, and MN NPs at different concentrations. (C) Live/dead cell analysis after treatments with MSA-2, NLG919, and MN NPs. Scale bars are 200 μm. (D) Cell uptake after incubation with ICG or ICG-NPs for 24 h. (E) CLSM image of 4T1 cells after incubation with free ICG or ICG-NPs at an equivalent concentration of ICG for 24 h. ICG was shown in green pseudo color. Nucleus and membrane were stained with 4′,6-diamidino-2-phenylindole (DAPI, blue) and 1,1′-dioctadecyl-3,3,3′,3′-tetramethylindocarbocyanineperchlorate (Dil, red), respectively. Scale bars are 20 μm. (F) Western blot analysis of IDO1 levels in 4T1 cells after different treatments. (G) The secretion level of Kyn in 4T1 cells after different treatments. Western blot analysis of protein levels in 4T1 cells (H) and DC2.4 cells (I) after different treatments. (J) IFN-β secretion of DC2.4 cells after different treatments. (K) Co-expression of CD86 and CD80 on bone marrow-derived dendritic cells (BMDCs). Results are expressed as mean ± SD (*n* = 3). *p < 0.05, **p < 0.01.

The cytotoxicity of MN NPs against 4T1 breast cancer cells was evaluated using the MTT assay. As shown in Fig. 3A, free MSA-2 just exhibited slight cytotoxicity even at high concentrations. In contrast, the cell viability after NLG919 and MN NP treatment reduced rapidly with increasing NLG919 concentration, and the inhibitory effect of NPs on cell viability was notably higher than that of the equivalent concentrations of free NLG919 (Fig. 3B). The cytotoxicity of MN NPs was further evaluated via a live/dead cell assay using propidium iodide (red) and calcein-AM (green) as stains for dead and live cells, respectively (Fig. 3C), where more cell death was observed after incubation with MN NPs compared with that in the other groups. Compared with free NLG919 and MSA-2, the improved cell cytotoxicity of MN NPs might be attributed to the effective cellular uptake. Indocyanine green (ICG) was encapsulated into MN NPs (ICG-NPs) for fluorescent labeling to investigate the cellular uptake of MN NPs. As shown in Fig. 3D and Figs. S8 and S9, the flow cytometry analysis showed that the 4T1 cell association of ICG-NPs was much higher than that of free ICG. CLSM images showed that the fluorescence signals of ICG in 4T1 cells treated with MN NPs were stronger than those in cells treated with free ICG, further demonstrating the enhanced cellular internalization of NPs (Fig. 3E). Meanwhile, the flow cytometry analysis demonstrated that the cellular uptake of MN NPs improved with the increase in incubation time (Fig. S10).

### IDO inhibition and STING activation in vitro

Blocking the IDO1 pathway to regulate the Trp/Kyn metabolism is an effective strategy to remodel the immunosuppressive TME. The protein levels of IDO1 in 4T1 cells after various treatments were analyzed using Western blotting to test the ability of MN NPs. As indicated in Fig. 3F, both NLG919 and MN NPs significantly down-regulated the IDO1 expression compared with the other groups. Specifically, the IDO1 expression was further reduced in the MN NP group compared with the NLG919 group, suggesting the effective blockade of the IDO pathway. Then, the reduced IDO1 further decreased the concentration of Kyn, an immunosuppressive metabolite derived from Trp. The ELISA results showed that MN NPs remarkably reduced the secretion of Kyn in 4T1 cells (Fig. 3G).

The activation of the STING signaling pathway is typically accompanied by the phosphorylation of TBK1 (p-TBK1), IRF3 (p-IRF3), and STING (p-STING), as well as the high production of type I IFNs and pro-inflammatory cytokines [43]. The protein expression in 4T1 cells was detected by Western blotting after different treatments to investigate the effects of MN NPs on the STING pathway. As shown in Fig. 3H, MN NPs significantly elevated the levels of p-TBK1 and p-STING, indicating potent STING activation. Meanwhile, the activation of the STING pathway in antigen-presenting cells (APCs) was also detected by Western blotting and ELISA. MSA-2 and MN NPs induced an obvious increase in the expression of p-TBK1, p-IRF3, and p-STING in DC2.4 cells (Fig. 3I). Moreover, ELISA results showed that MN NPs dramatically enhanced the secretion of IFN-β (the hallmark of STING activity) in DC2.4 cells among all groups (*P* < 0.05) (Fig. 3J), further confirming the STING pathway activation.

Increasing evidence has demonstrated that STING activation can develop the crosstalk between tumors and nearby immune cells via the released cytokines [44]. Also, the STING pathway in APCs directly triggered efficient cellular activation, including DC maturation and macrophage polarization [43]. The maturation of bone marrow-derived dendritic cells (BMDCs) was investigated by flow cytometry to investigate the role of MN NPs in immune activation. As shown in Fig. 3K and Figs. S11 and S12, NLG919 induced negligible expression of costimulatory molecules and failed to activate BMDC maturation. In contrast, MSA-2 and MN NPs induced higher levels of CD86 and CD80 than those in the phosphate-buffered saline (PBS) and NLG919 groups, which was attributed to the adjuvant effect of MSA-2. Additionally, the increased secretion of tumor necrosis factor α (TNF-α) and interleukin 6 (IL-6) in BMDCs also indicated the robust immune activation potential of the MN NPs (Fig. S13). Overall, the results suggested that MN NPs could efficiently reverse the immunosuppressive TME by regulating the Kyn metabolism, activating the STING pathway, and inducing DC maturation, thereby triggering a robust antitumor immune response.

### Biodistribution and antitumor performance in vivo

**Fig. 4.**
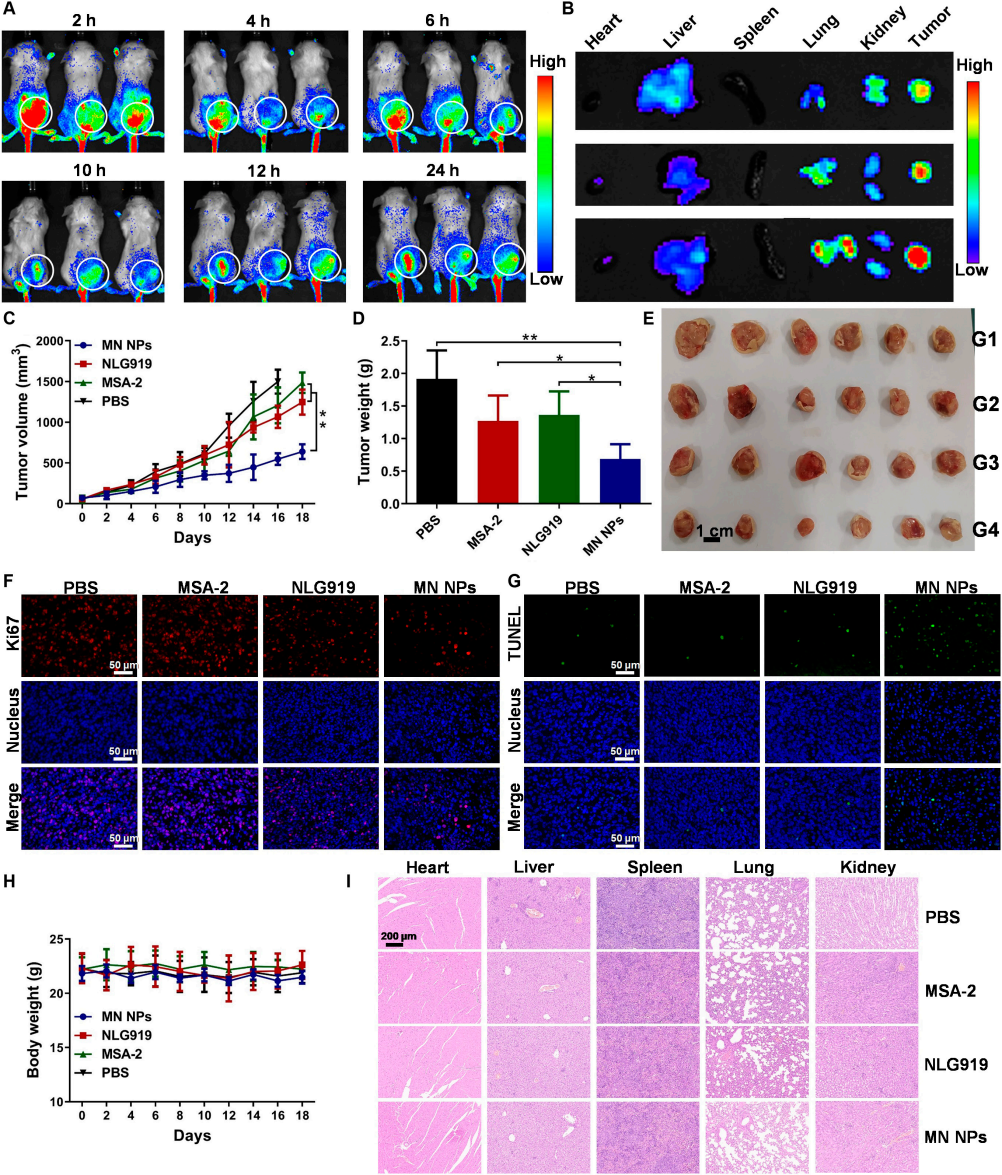
Biodistribution and antitumor performance in vivo. (A) Distribution of fluorescently labeled MN NPs in vivo. (B) Ex vivo fluorescence images of the main organs and tumors 24 h post-injection of MN NPs. (C) Tumor growth curves of mice. Weights (D) and photographs (E) of tumors harvested from mice. Ki67 (F) and TUNEL (G) staining images of tumors, where red, blue, and green dots represent tumor cell proliferation, nuclei, and apoptotic cells, respectively. (H) Changes in body weight of mice. (I) H&E staining images of major organs. Scale bars are 200 μm.

A major obstacle hindering the application of therapeutic molecules is insufficient tumor accumulation, resulting in unsatisfactory therapeutic outcomes and severe side effects [45,46]. Fluorescence imaging was employed to examine the biodistribution of fluorescently labeled MN NPs in 4T1 tumor-bearing mice at the determined time points to assess the accumulation of MN NPs within the tumors. As shown in Fig. 4A, obvious fluorescence signals were detected from tumors within 24 h after the intravenous injection of MN NPs. The accumulation of MN NPs in tumors exhibited a time-dependent pattern, with the highest fluorescence intensity observed 2 h post-injection (Fig. S14). Ex vivo fluorescence images of major organs and tumors obtained 24 h post-injection further validated the substantial accumulation of MN NPs in tumors (Fig. 4B and Fig. S15).

Then, the effectiveness of MN NPs in treating 4T1 tumor-bearing mice was examined. As illustrated in Fig. 4C, free NLG919 or MSA-2 only slightly decreased the growth of tumors compared with that in the PBS group. Notably, MN NPs significantly inhibited tumor growth compared with free NLG919 or MSA-2, which could be attributed to the high tumor accumulation and improved immunotherapy. The differences in average weight and size of the tumors 18 days after treatment also demonstrated the robust antitumor effect of MN NPs (Fig. 4D and E). The antitumor efficacy was further investigated by the immunohistochemical staining of Ki-67 and terminal deoxynucleotidyl transferase dUTP nick-end labeling (TUNEL). Ki67 images indicated obvious inhibition of tumor proliferation after treatment with MN NPs, confirming their superior tumor suppression (Fig. 4F). The TUNEL images of tumor slices suggested that MN NPs induced the strongest cellular apoptosis in all groups (Fig. 4G). The safety of different formulations was assessed by tracking alterations in the mouse body weight, blood biochemistry, and pathological section. No obvious differences in body weight were found between the groups (Fig. 4H). As shown in Fig. 4I, hematoxylin and eosin (H&E) staining of major organs showed no histopathologic damage in all groups. Furthermore, the levels of lactate dehydrogenase, alanine transaminase, aspartate transaminase, and alkaline phosphatase in blood were similar in all groups (Fig. S16), indicating that MN NPs exhibited good biocompatibility.

### Immune responses after treatment with NPs

**Fig. 5.**
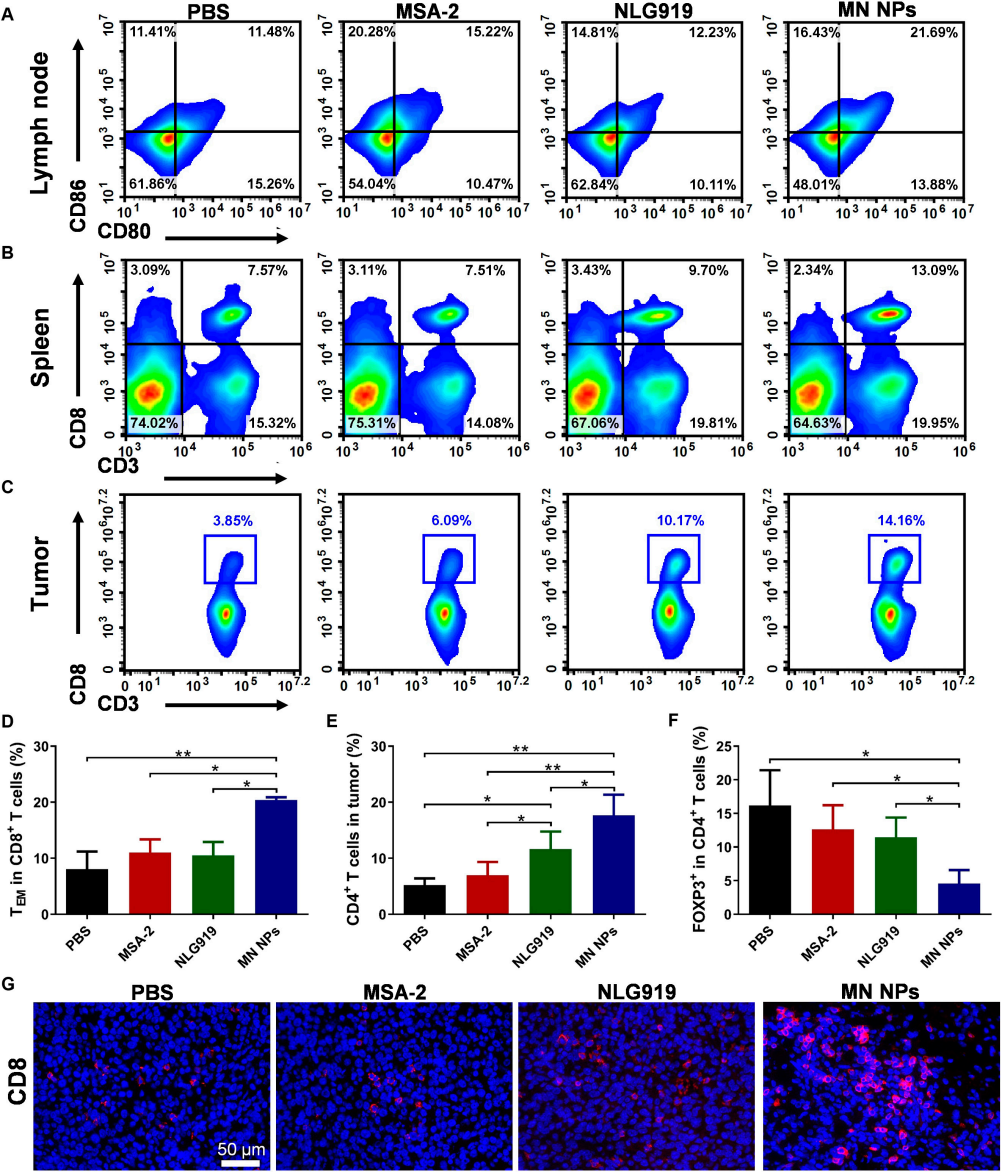
Immune responses induced by NPs. Representative flow cytometry analysis of DC maturation in tumor-draining LNs (A), CD3^+^CD8^+^ T cells in the spleen (B), and tumors (C). The frequency of effector memory T cells (T_EM_, CD8^+^CD44^+^CD62L^−^) in the spleen (D), CD3^+^CD4^+^ in tumors (E), and Tregs (CD4^+^Foxp3^+^) in tumors (F). (G) Immunofluorescence staining images of CD8^+^ T cells in tumors. Results are expressed as mean ± SD (*n* = 6). *p < 0.05, **p < 0.01.

Following the study of tumor suppression in vivo and immune activation in vitro, the in vivo immune activation induced by MN NPs was also evaluated. As shown in Fig. 5A and Fig. S17, MSA-2 and MN NPs enhanced DC maturation in tumor-draining lymph nodes (LNs) compared with that in the PBS group. In particular, the MN NPs exhibited the best ability to promote DC maturation among all groups, aligning with the in vitro results. The proportion of T helper cells (CD3^+^CD4^+^ T cells) and cytotoxic T lymphocytes (CD3^+^CD8^+^ T cells) in the spleen was analyzed to investigate systemic immunoactivation. The flow cytometry results demonstrated that MN NPs significantly increased the percentages of CD8^+^ (Fig. 5B and Figs. S18 and S19) and CD4^+^ T cells (Figs. S20 and S21), indicating systemic antitumor immunity. The immune memory response is crucial in eradicating secondary infections and metastatic tumor cells [47]. The percentage of memory T cells in splenocytes was quantified by flow cytometry to verify whether MN NPs had this effect. An important elevation in the quantity of effector memory T cells (T_EM_, CD8^+^CD44^+^CD62L^−^) was observed in the MN NP group (Fig. 5D and Fig. S22), suggesting their ability for long-term immune memory.

The infiltration of CD8^+^ and CD4^+^ T cells into the tumor, which is crucial for effective tumor immunotherapy, was explored by flow cytometry after different treatments. The MN NPs elicited the highest frequency of CD8^+^ (Fig. 5C and Figs. S23 and S24) and CD4^+^ T cell (Fig. 5E and Fig. S25) infiltration in tumors. Meanwhile, the number of immunosuppressive Tregs (CD4^+^Foxp3^+^) decreased after the treatments (Fig. 5F and Fig. S26). The MN NPs were more effective in decreasing the number of Tregs compared with the NLG919 group. As an indicator of antitumor immunity, the intertumoral ratio of effector T cells to Tregs (CD8^+^ T cells/Tregs) was also measured. The maximum ratio of effector T cells and Tregs was observed in the MN NP group compared with the other groups (Fig. S27), which further confirmed the strong T-cell-mediated antitumor immune responses of NPs. This was attributed to the amplification of immunoactivities by combining STING activation and the amelioration of the immunosuppressive TME following the blockade of the IDO1 pathway. Following that, the tumor tissues were obtained and analyzed by immunofluorescence staining. MN NPs resulted in a higher number of CD8^+^ T cells in the tumors due to the combination of enhanced immunoactivities and the attenuated immunosuppressive TME (Fig. 5G). Overall, these data demonstrated that MN NPs could induce robust antitumor immunity by enhancing DC maturation, facilitating T-cell infiltration, and influencing the T-cell composition. This, in turn, had the potential to prevent metastasis.

### Transcriptomic analysis

**Fig. 6.**
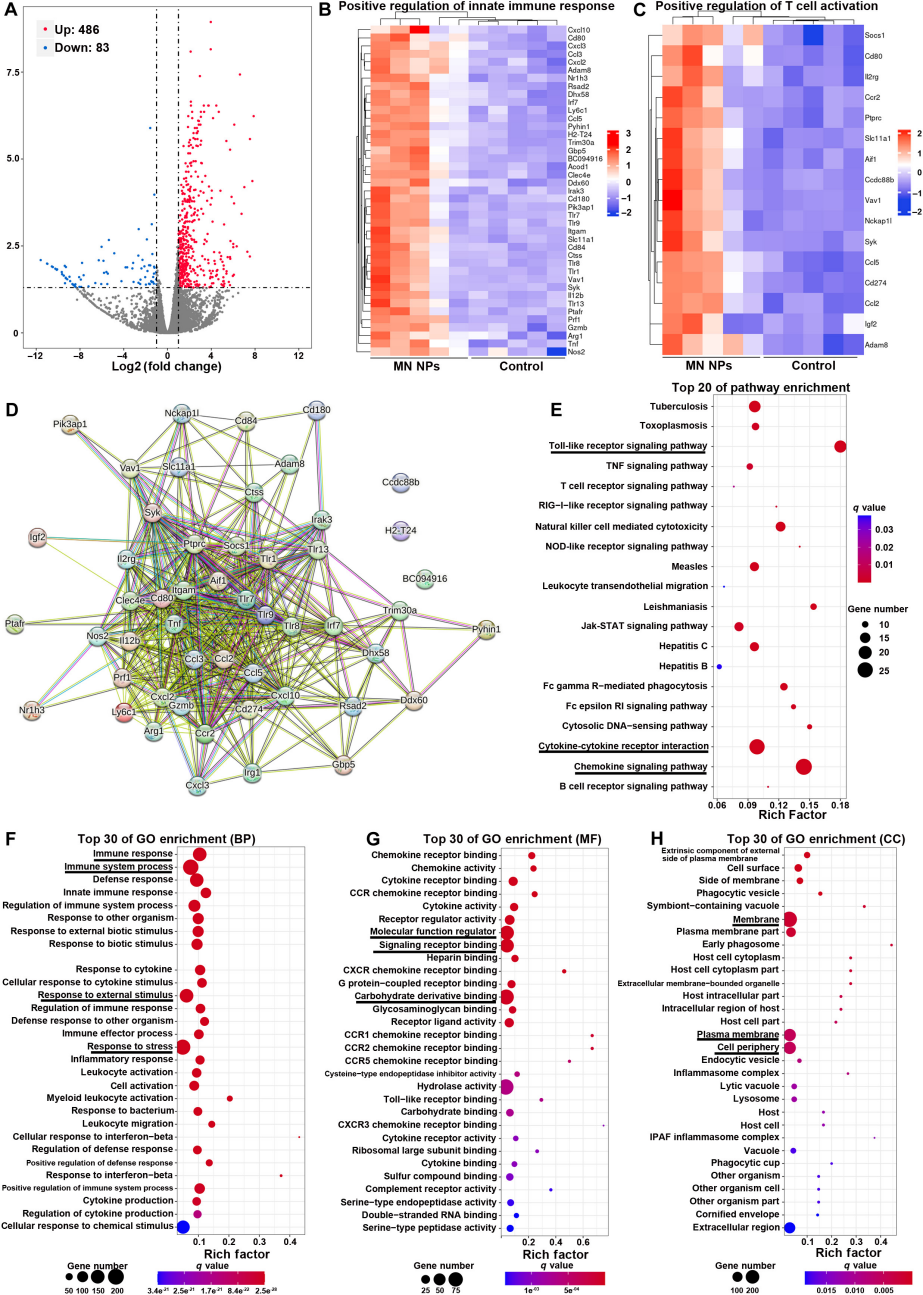
Transcriptomic analysis of tumor tissues derived from mice after different treatments. (A) Volcano plot showing DEGs in the control and MN NP groups. Heat map displaying the differentially expressed immune-related genes involved in the positive regulation of innate immune response (B) and positive regulation of T-cell activation (C) in tumors after the treatment. Red and blue represent up-regulation and down-regulation, respectively (*n* = 5). (D) Functional interaction networks of the immune-related genes. (E) KEGG enrichment analysis of the pathways involved in the biological effects induced by MN NPs. Up-regulated biological processes (F), molecular functions (G), and cell components (H) in tumor tissues based on GO annotation analysis.

The immune state of tumor tissues from mice after the treatment with MN NPs was also investigated by transcriptomic analysis. The heat map of the differentially expressed genes (DEGs) exhibited a notable difference between the control (PBS) and MN NP groups (Fig. S28). The DEGs with a threshold fold change above 2 and a *P* value below 0.05 between the control (PBS) and MN NP groups are shown in the volcano plot (Fig. 6A). A total of 488 genes were found to be up-regulated, and 83 genes were down-regulated in total. The immune-related genes that were differentially expressed in the categories of “positive regulation of innate immune response” and “positive regulation of T-cell activation” were further selected to assess the innate immune response and T-cell activation in tumors (Fig. 6B and C). As shown in the functional associated networks (Fig. 6D), a majority of the immune-related genes exhibited physical interactions with each other. Kyoto Encyclopedia of Genes and Genomes (KEGG) enrichment analysis revealed that the up-regulated genes after the treatment with MN NPs were enriched in cytokine signaling pathways and immune response–associated signaling pathways (e.g., cytokine–cytokine receptor interactions), chemokine signaling pathway, Toll-like receptor signaling pathway, and NK cell–mediated cytotoxicity (Fig. 6E). The DEGs were categorized into biological process, cell components, and molecular functions based on their functions using Gene Ontology (GO) annotation analysis (Fig. 6F to H). GO enrichment analysis indicated that most up-regulated genes played roles in processes related to the immune system, immune responses, response to various stimuli, binding, biological regulation, and cellular components.

### Inhibition of metastatic tumor growth

**Fig. 7.**
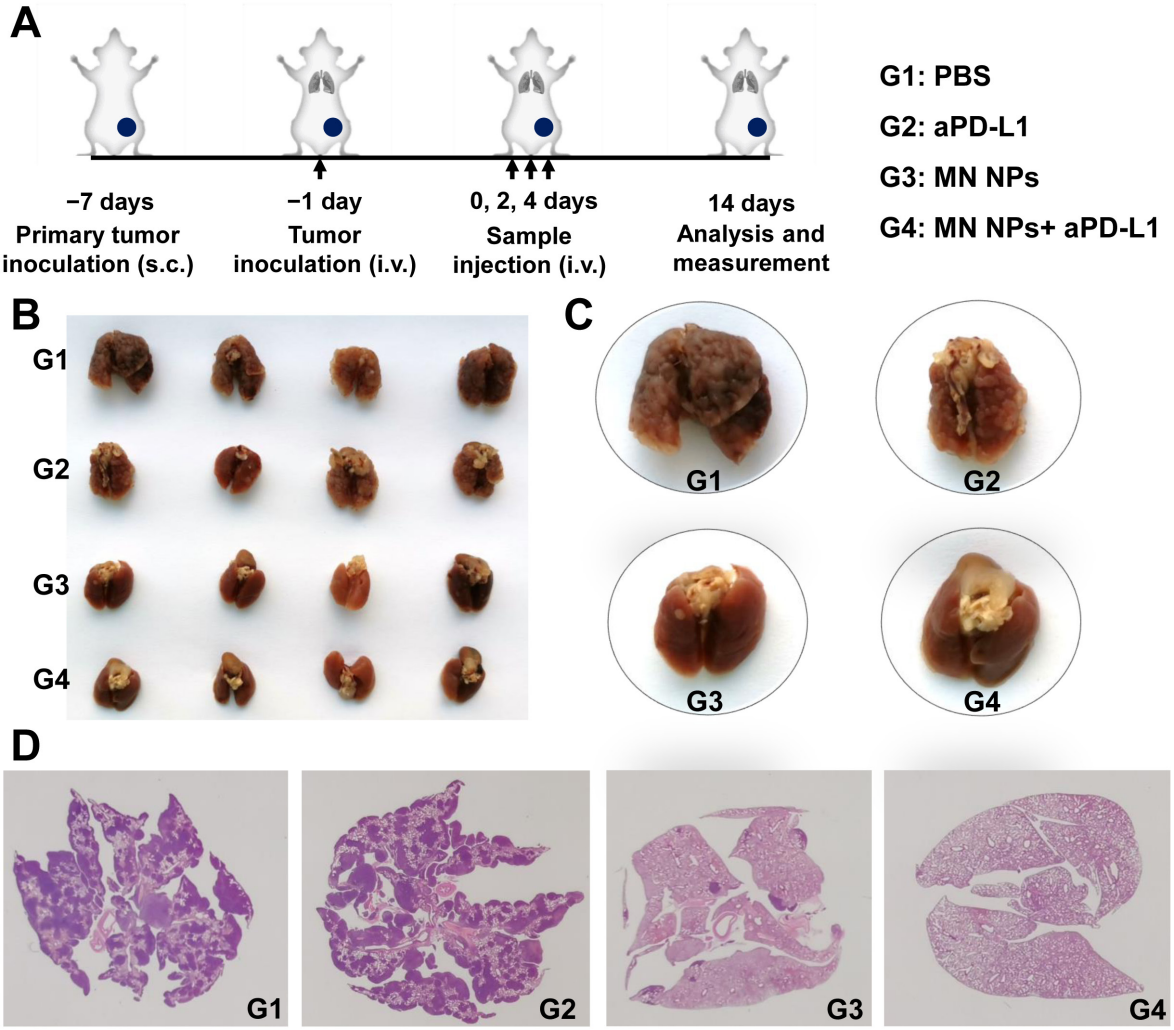
Inhibition of metastatic tumor growth by NPs. (A) Schematic illustration of the experimental procedure involving metastatic tumors. Representative photographs (B), magnified photographs (C), and H&E staining images (D) of the lungs post-treatment.

The anti-metastatic effect of the MN NPs, in combination with the anti-PD-L1 antibody (aPD-L1) immune checkpoint inhibitor, was investigated. A mouse model of lung metastasis was established by subcutaneously and intravenously injecting 4T1 cells into mice at different time points, following the procedure described in Fig. 7A. After different treatments, lung tumor metastasis was observed to evaluate the therapeutic effect. The mice in the PBS group manifested the most severe lung metastasis, with tumors expanding throughout the lung (Fig. 7B and C). In contrast, MN NPs, especially MN NPs combined with aPD-L1, significantly inhibited metastatic tumor growth. Further, H&E staining showed dense tumor nodules distributed in the lungs in the PBS and aPD-L1 groups. In contrast, the number of these nodules obviously reduced after MN NP treatment, especially when combined with aPD-L1 (Fig. 7D), further confirming the efficacy of the combined MN NPs/ICB strategy in suppressing metastatic tumor growth.

### Antitumor efficacy in a mouse melanoma tumor model

**Fig. 8.**
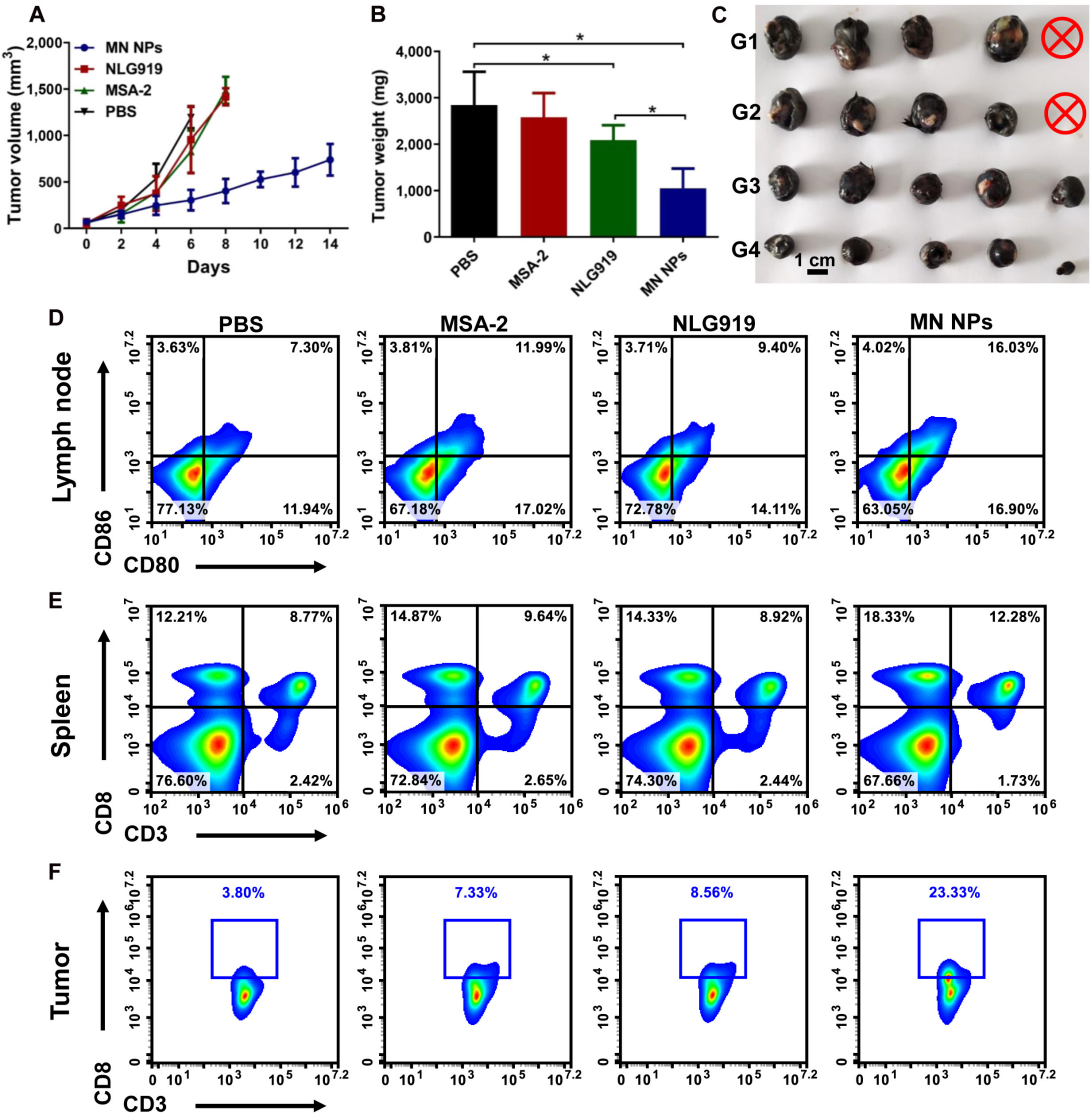
Antitumor efficacy in the B16 tumor model. (A) Tumor growth curves in mice bearing B16 tumors after various treatments. Weights (B) and photographs (C) of tumors harvested from mice bearing B16 tumors on day 14 after different treatments. Representative flow cytometry analysis of DC maturation in tumor-draining LNs (D), CD3^+^CD8^+^ T cells in the spleen (E), and tumors (F). Results are expressed as mean ± SD (*n* = 5). *p < 0.05.

The therapeutic antitumor efficacy was further investigated in a mouse B16 melanoma tumor model established using C57BL/6 mice to evaluate the generality of MN NPs in inhibiting solid tumors. Similar to the 4T1 breast tumor model, the tumor growth in the MN NP group was significantly slower than that in other groups (Fig. 8A). The average weight and size of the tumors 14 days after treatment further validated the good tumor-suppressive ability of MN NPs (Fig. 8B and C). Then, the immune activation triggered by MN NPs in the tumor-draining LNs, spleen, and tumors was assessed using flow cytometry. As shown in Fig. 8D and Fig. S29, MN NP treatment remarkably promoted DC maturation in LNs compared with that in other groups, which was consistent with the results of the 4T1 model. Moreover, a substantial rise in the population of CD8^+^ T cells in both the spleen and tumors was observed in the MN NP group, further proving their capacity for systemic and local immunoactivation (Fig. 8E and F and Figs. S30 and S31). Meanwhile, MN NP treatment significantly decreased the number of immunosuppressive Tregs in tumors compared with other groups (Figs. S32 and S33). The biocompatibility of each formulation was also verified by the nondecreased mouse body weight across all treatment groups (Fig. S34). These results suggested that MN NPs had the potential to combat different solid tumors by synergistically modulating the immune microenvironment.

## Discussion

The altered metabolic pathways in tumor cells create a suppressive TME that suppresses the immune response and impedes the effectiveness of various cancer treatments [5,6]. Specifically, metabolites such as glucose, lactate, amino acids, and adenosine facilitate the growth of immunosuppressive cells, including tumor-associated macrophages, Tregs, and myeloid-derived suppressor cells, leading to resistance to chemotherapy, phototherapy, and immunotherapy. Therefore, regulating the immunosuppressive metabolic TME, such as the production of suppressive metabolites, hypoxia, and acidic pH, has emerged as a promising strategy to enhance antitumor effectiveness.

Energy serves as the foundation for cellular activities. Unlike normal cells that use oxidative phosphorylation for energy production, tumor cells predominantly utilize glycolysis to generate energy even in aerobic conditions [7,8]. Given this, inhibiting glycolysis to disrupt the energy supply of tumor cells is a widely studied approach for metabolic intervention. Recently, a variety of biomaterials have been developed to suppress glycolysis for tumor combinational immunotherapy, including metal-organic frameworks, nanomicelles, and mesoporous silica NPs [48,49]. Among them, stimuli-responsive nanomedicine, triggered by endogenous factors (such as low pH, overexpressed enzymes, high levels of redox agents, and ATP) and/or exogenous stimuli (such as light, radiation, ultrasound, and temperature), have garnered increased attention for tumor-specific treatments [50].

Additionally, amino acids such as glutamine, methionine, Trp, and Kyn are critical factors for cellular function and play an equally important role in generating energy [6]. In this study, we have demonstrated that the synergistic regulation of amino acid metabolism and STING pathway activation can effectively inhibit tumor growth. Therapeutic molecules (NLG919 and MSA-2) are directly utilized as building blocks to construct NPs, resulting in significantly improved drug-loading efficiency and biological stability. The suitable size of these self-assembled NPs promoted cellular uptake and tumor accumulation, effectively addressing the key challenges in translating hydrophobic drugs to clinical use, such as rapid clearance and inadequate tissue absorption [45,46]. The enhanced therapeutic effectiveness against various solid tumors indicates that these self-assembled NPs can successfully deliver therapeutic drugs to tumor sites and synergistically regulate the immune microenvironment to inhibit tumor growth. However, we have only investigated how remodeling Trp/Kyn metabolism enhances the effectiveness of tumor immunotherapy. In the future, we will further investigate the effect of regulating different types of metabolites on tumor immunity and combine them with STING and toll-like receptor (TLR) agonists to enhance treatment effectiveness. Moreover, targeting molecules should be introduced on the surface of NPs to further enhance their accumulation at tumor sites, thereby improving their bioavailability.

### Conclusion

MN NPs were successfully fabricated via metal coordination-driven assembly for remodeling the immune microenvironment. The MN NPs accumulated in tumors and were efficiently taken up by tumor cells, avoiding systemic diffusion of the therapeutic molecules and thereby improving their potency. Importantly, the obtained MN NPs exhibited excellent capacity for amino acid metabolic modulation and STING stimulation, transforming immunosuppressive tumors into immunogenic tumors. Thus, a high degree of DC maturation, tumor infiltration of effector immune cells, Treg reduction, and immunological memory was achieved using the MN NPs, which, in turn, resulted in the inhibition of tumor growth and tumor metastasis. Overall, this study provided a novel paradigm for enhancing tumor immunotherapy through synergistic amino acid metabolism and STING pathway activation.

#### Ethical Approval

All animal procedures were conducted in compliance with the Guidelines for the Care and Use of Laboratory Animals of Shandong First Medical University & Shandong Academy of Medical Sciences.

## Supplementary Material

20240704-1

## Data Availability

The data used to support the findings of this work are available from the corresponding authors upon request.
