# Erratum to “Amino Acid Metabolism-Regulated Nanomedicine for Enhanced Tumor Immunotherapy through Synergistic Regulation of Immune Microenvironment”

**DOI:** 10.34133/bmr.0352

**Published:** 2026-04-21

**Authors:** Xiuying Duan, Yilei Zhao, Houyang Hu, Xuechun Wang, Jie Yan, Songyan Li, Yueying Zhang, Jianwei Jiao, Guiqiang Zhang

**Affiliations:** ^1^ Medical Science and Technology Innovation Center, Shandong First Medical University & Shandong Academy of Medical Sciences, Jinan, Shandong 250117, China.; ^2^ School of Life Sciences, Shandong First Medical University & Shandong Academy of Medical Sciences, Jinan, Shandong 250117, China.; ^3^ School of Clinical and Basic Medical Sciences, Shandong First Medical University & Shandong Academy of Medical Sciences, Jinan, Shandong 250117, China.; ^4^ State Key Laboratory of Stem Cell and Reproductive Biology, Institute of Zoology, Chinese Academy of Sciences, Beijing 100101, China.

In the article “Amino Acid Metabolism-Regulated Nanomedicine for Enhanced Tumor Immunotherapy through Synergistic Regulation of Immune Microenvironment,” an error has been identified in Fig. 4B and Fig. 5G [1]. During the revision process, *ex vivo* fluorescence image of one mouse and the immunofluorescence staining image of CD8^+^ T cells in MN NPs were inadvertently misplaced. The corrected figures are below and have also been updated in the original HTML and PDF. The authors apologize for the inconvenience and assure readers that this correction does not affect the results, interpretations, or conclusions of the study.

**Fig. 4.**
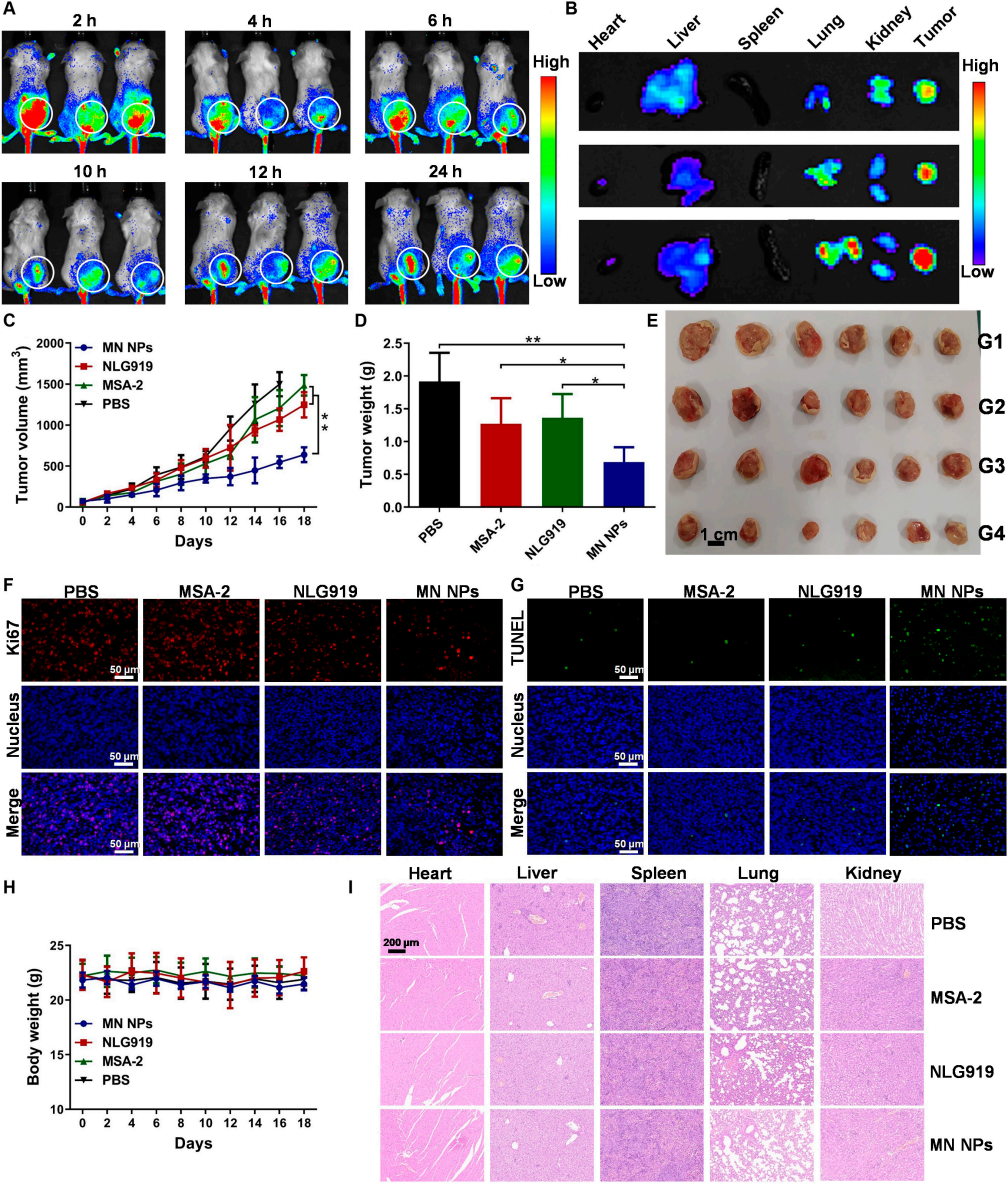
Biodistribution and antitumor performance in vivo. (A) Distribution of fluorescently labeled MN NPs in vivo. (B) Ex vivo fluorescence images of the main organs and tumors 24 h post-injection of MN NPs. (C) Tumor growth curves of mice. Weights (D) and photographs (E) of tumors harvested from mice. Ki67 (F) and TUNEL (G) staining images of tumors, where red, blue, and green dots represent tumor cell proliferation, nuclei, and apoptotic cells, respectively. (H) Changes in body weight of mice. (I) H&E staining images of major organs. Scale bars are 200 μm. (For interpretation of the references to colour in this figure legend, the reader is referred to the web version of this article.)

**Fig. 5.**
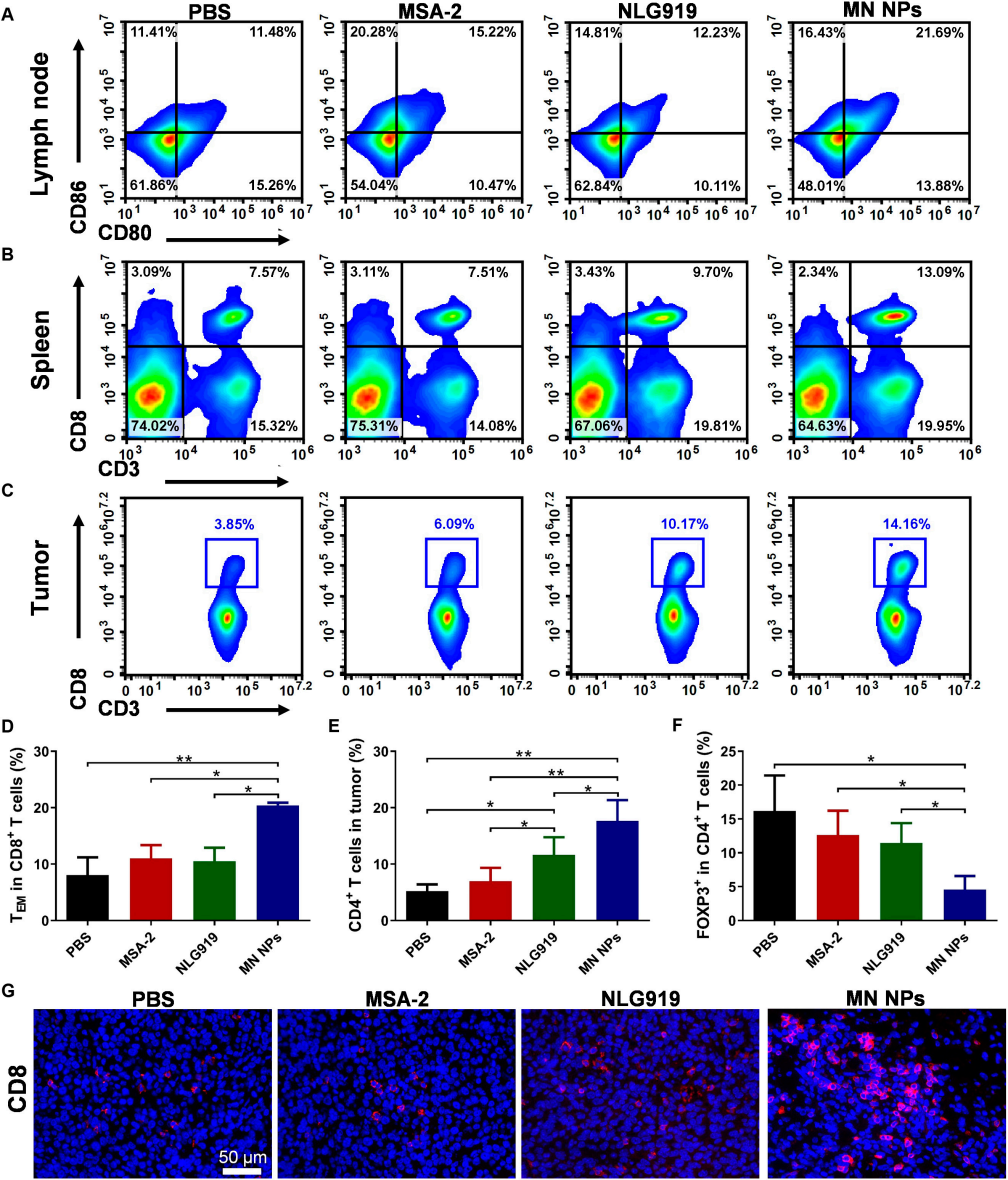
Immune responses induced by NPs. Representative flow cytometry analysis of DC maturation in tumor-draining LNs (A), CD3^+^CD8^+^ T cells in the spleen (B), and tumors (C). The frequency of effector memory T cells (TEM, CD8^+^CD44^+^CD62L^-^) in the spleen (D), CD3^+^CD4^+^ in tumors (E), and Tregs (CD4^+^Foxp3^+^) in tumors (F). (G) Immunofluorescence staining images of CD8^+^ T cells in tumors. Results are expressed as mean ± SD (*n* = 6). *p < 0.05, **p < 0.01.
