# Supplementary material for: Amino Acid Metabolism-Regulated Nanomedicine for Enhanced Tumor Immunotherapy through Synergistic Regulation of Immune Microenvironment
Source: Biomater Res. 2024 Jul 4;28:0048. doi: 10.34133/bmr.0048 (PMC11223770; doi:10.34133/bmr.0048)
Supplement: Supplementary 1 — Figs. S1 to S34 [file bmr.0048.f1.docx]

**Supporting information**

Amino acid metabolism-regulated nanomedicine for enhanced tumor immunotherapy through synergistic regulation of immune microenvironment

Xiuying Duan,^a,b^ Yilei Zhao^a^, Houyang Hu^a^, Xuechun Wang^a^, Jie Yan^a^, Songyan Li^a^, Yueying Zhang^d,^*, Jianwei Jiao,^c,^* and Guiqiang Zhang^a,^*

^a^Medical Science and Technology Innovation Center, Shandong First Medical University & Shandong Academy of Medical Sciences, Jinan, Shandong 250117, China.

^b^School of Life Sciences, Shandong First Medical University & Shandong Academy of Medical Sciences, Jinan, Shandong 250117, China.

^c^State Key Laboratory of Stem Cell and Reproductive Biology, Institute of Zoology, Chinese Academy of Sciences, Beijing 100101, China.

^d^School of Clinical and Basic Medical Sciences, Shandong First Medical University & Shandong Academy of Medical Sciences, Jinan, Shandong 250117, China.

*To whom correspondence should be addressed:

E-mail: gqzhang2018@163.com; jwjiao@ioz.ac.cn; zhangyueying@sdfmu.edu.cn

Full address: No.6699 Qingdao Road, Jinan, 250117, P.R. China.


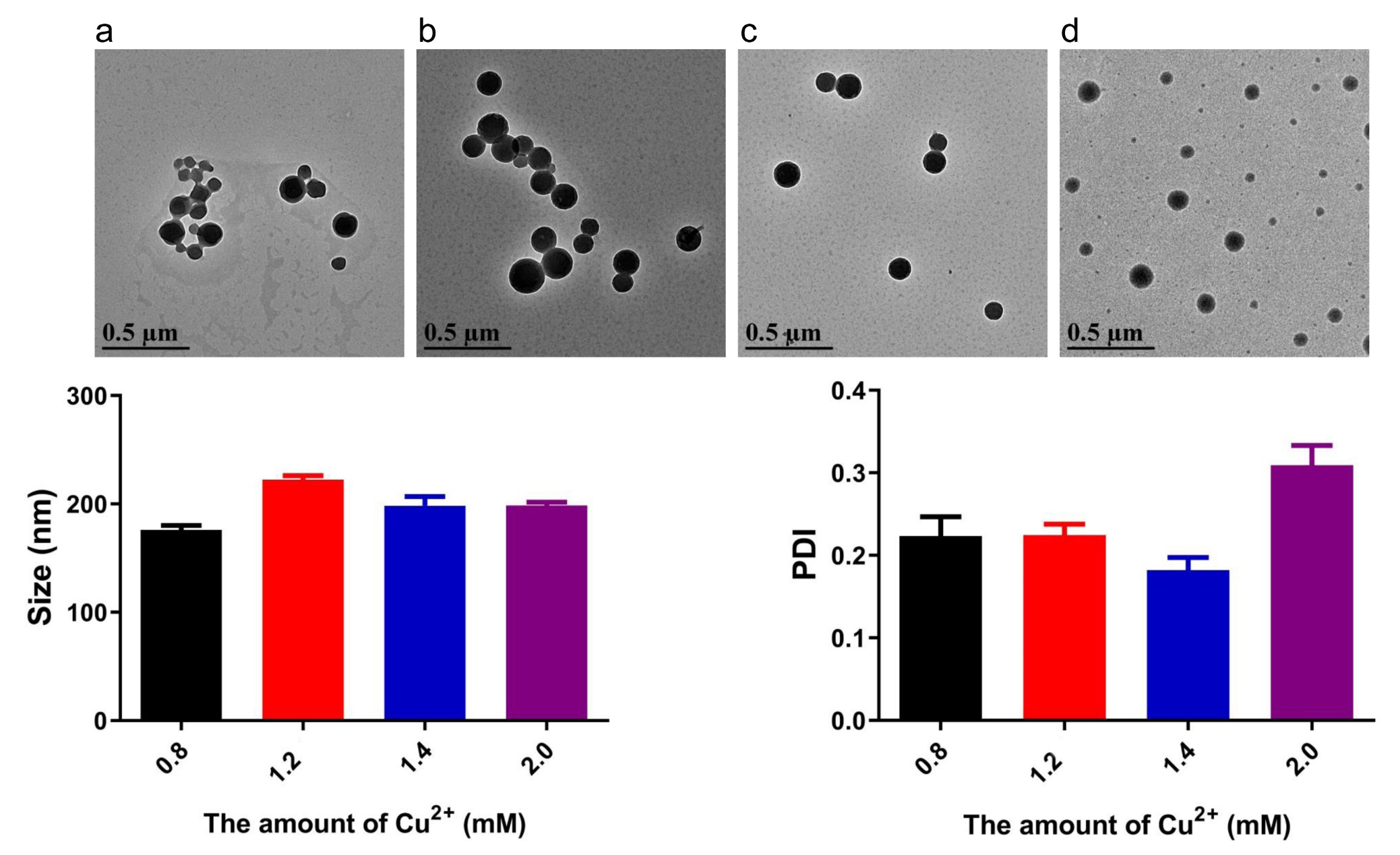


**Figure S1.** TEM images and the corresponding size and PDI distributions of the NPs. The Cu^2+^ amount was 0.8 mM (a), 1.2 mM (b), 1.4 mM (c), and 2.0 mM (d), respectively.


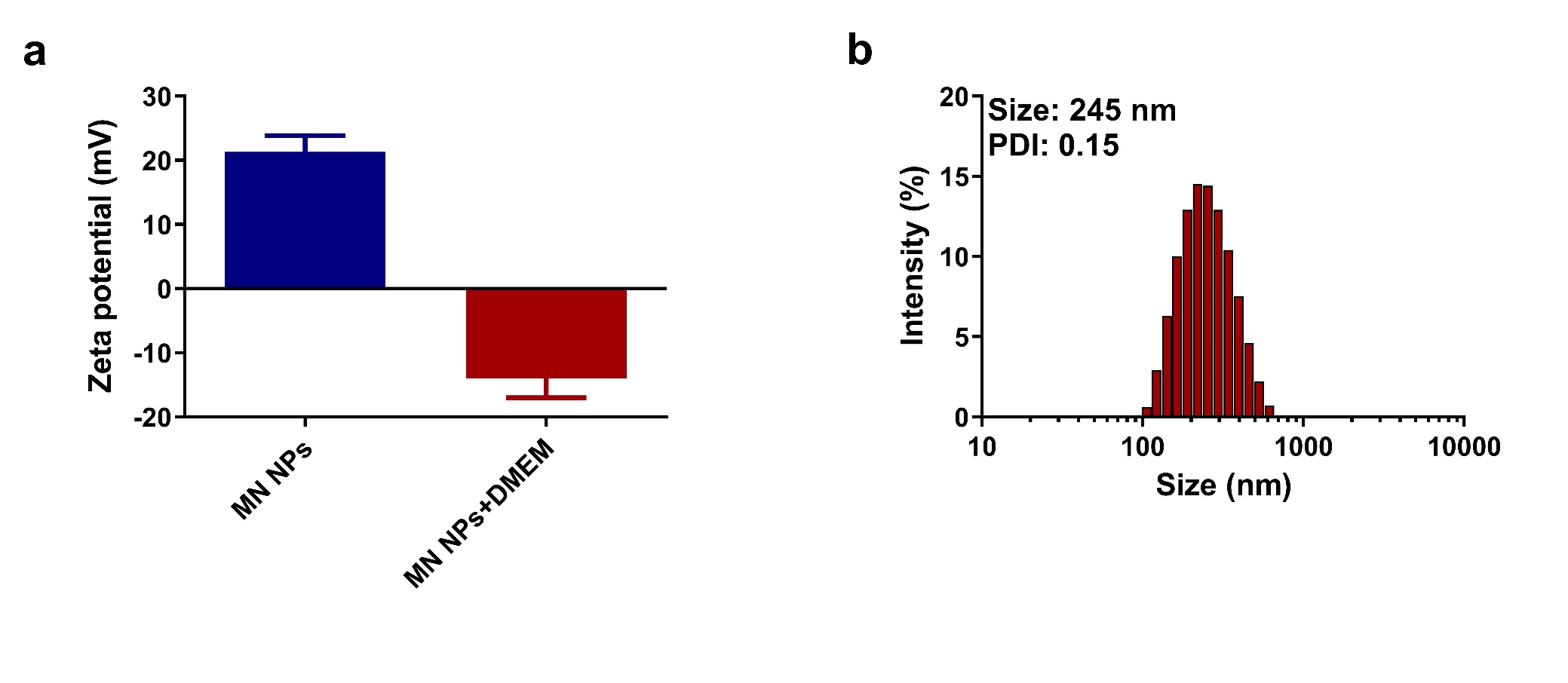


**Figure S2.** Zeta potential (a) and size distribution (b) of the assembled NPs in DMEM containing 10% FBS.


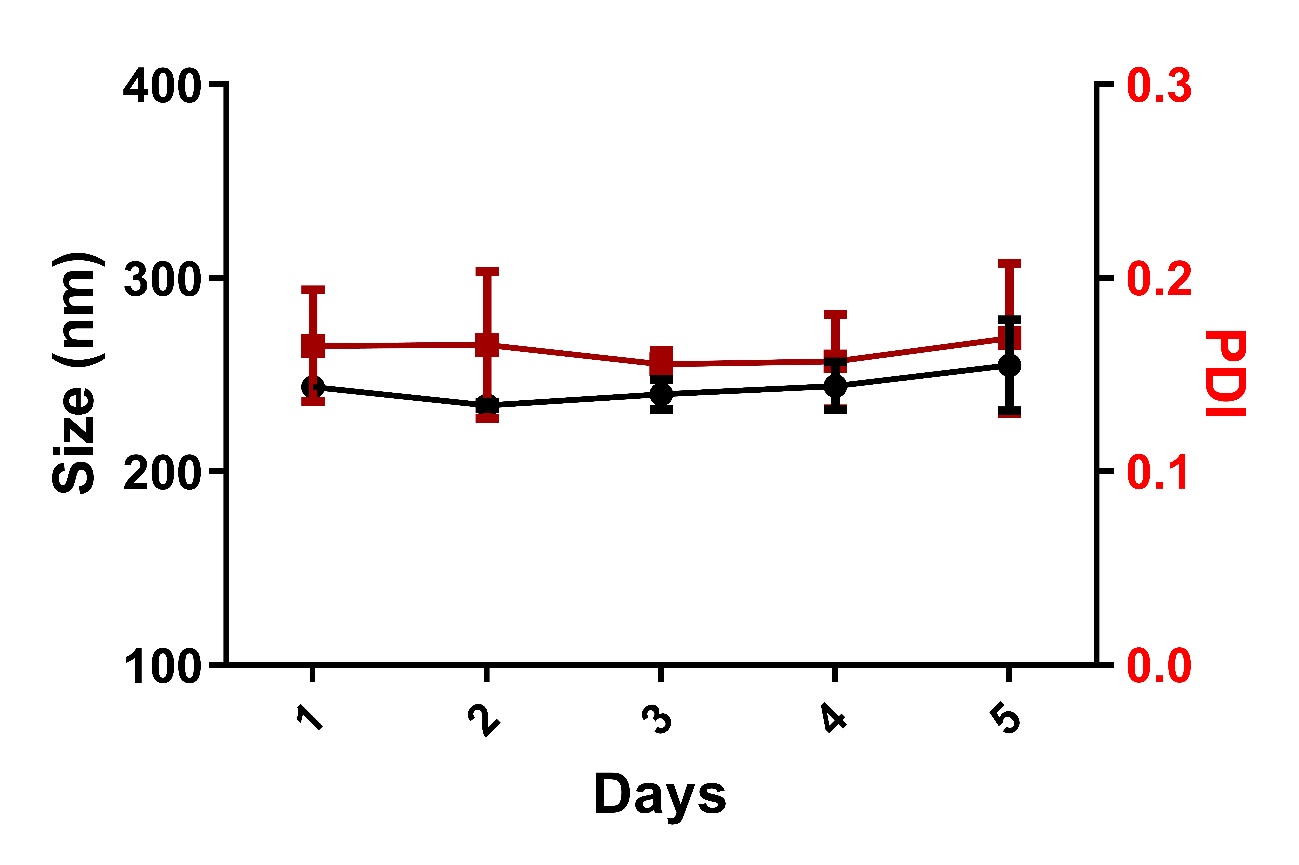


**Figure S3.** Colloidal stability of MN NPs in DMEM containing 10% FBS.


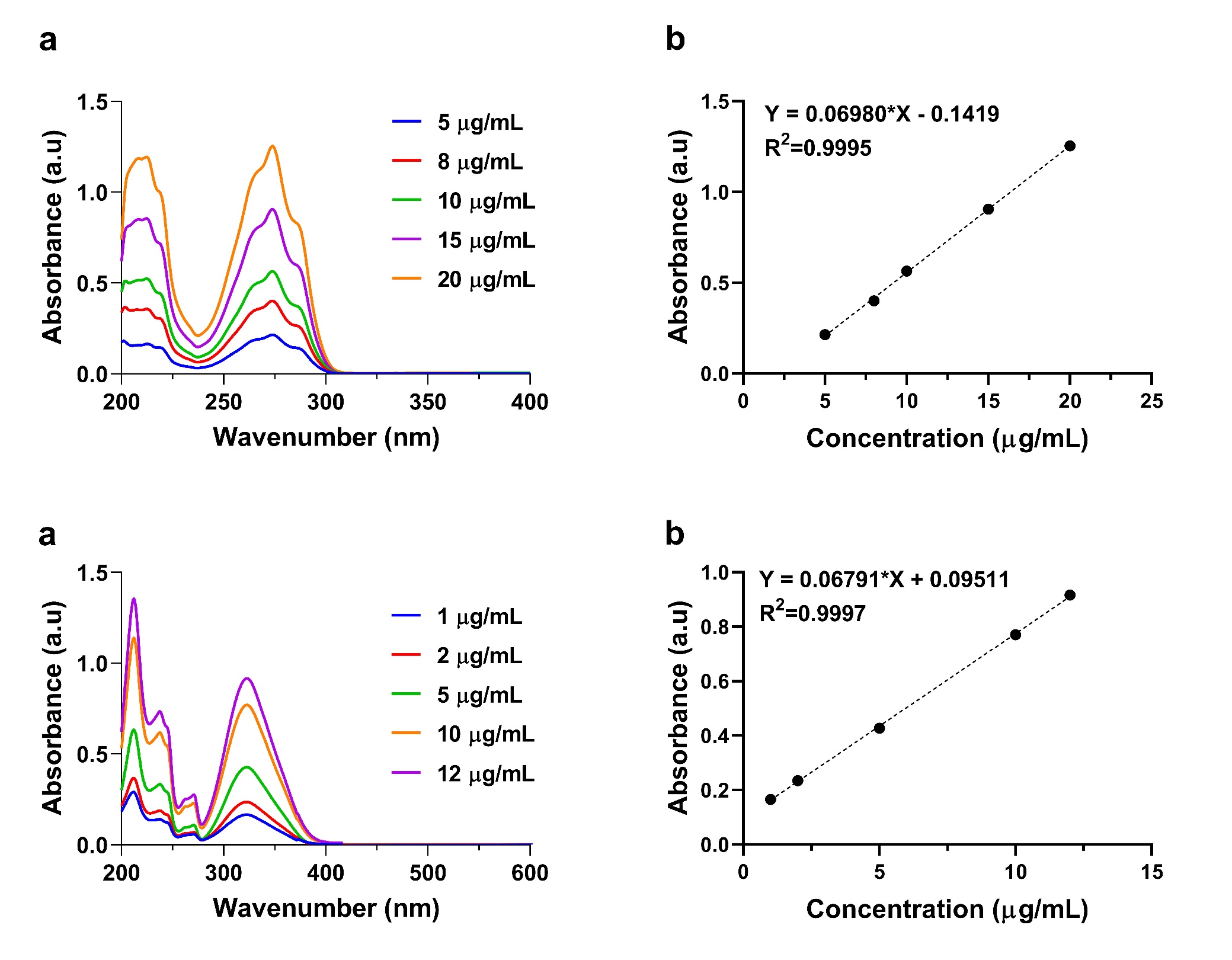


**Figure S4.** UV-Vis absorbance spectra (a) and standard curve (b) of NLG919.


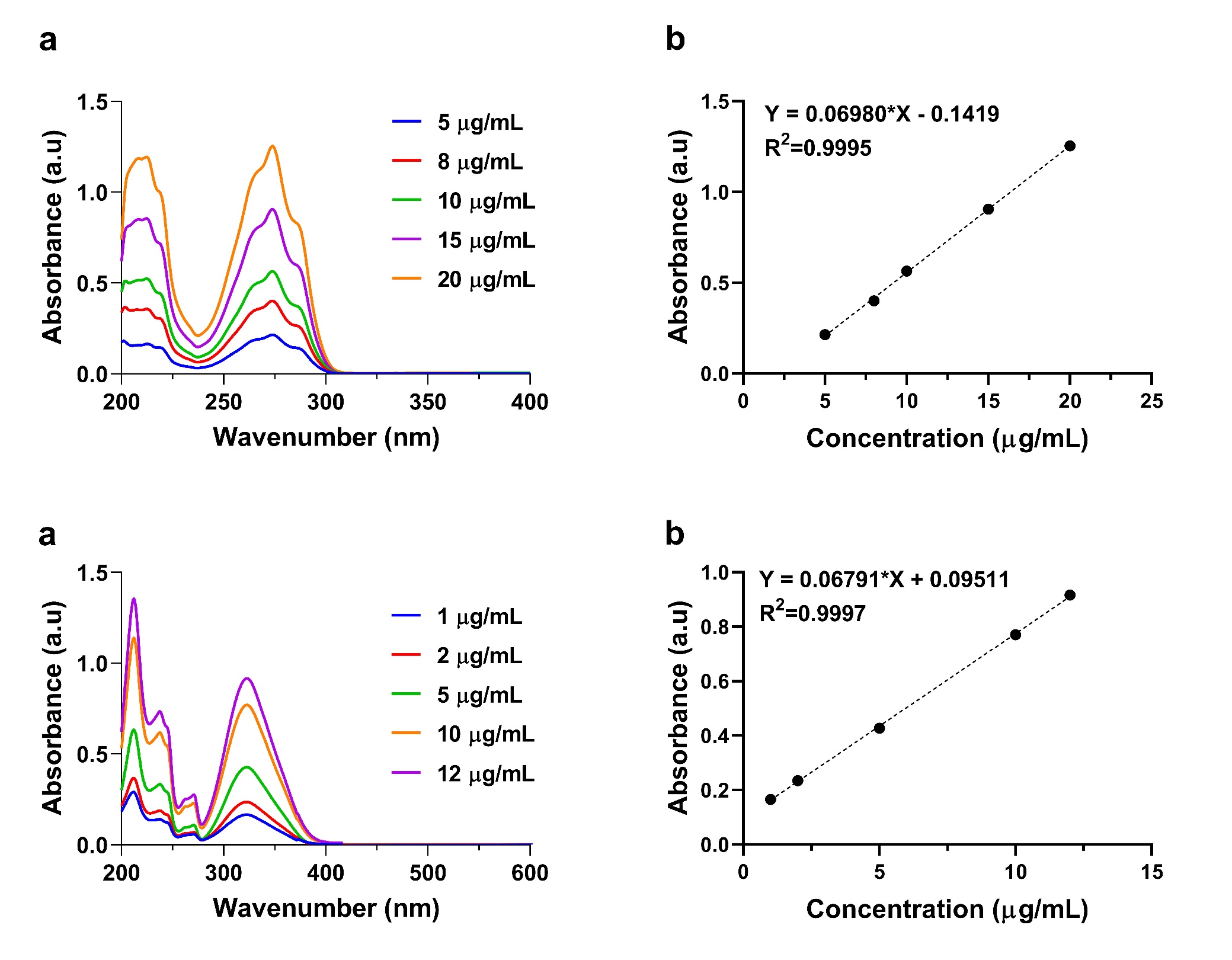


**Figure S5.** UV-Vis absorbance spectra (a) and standard curve (b) of MSA-2.


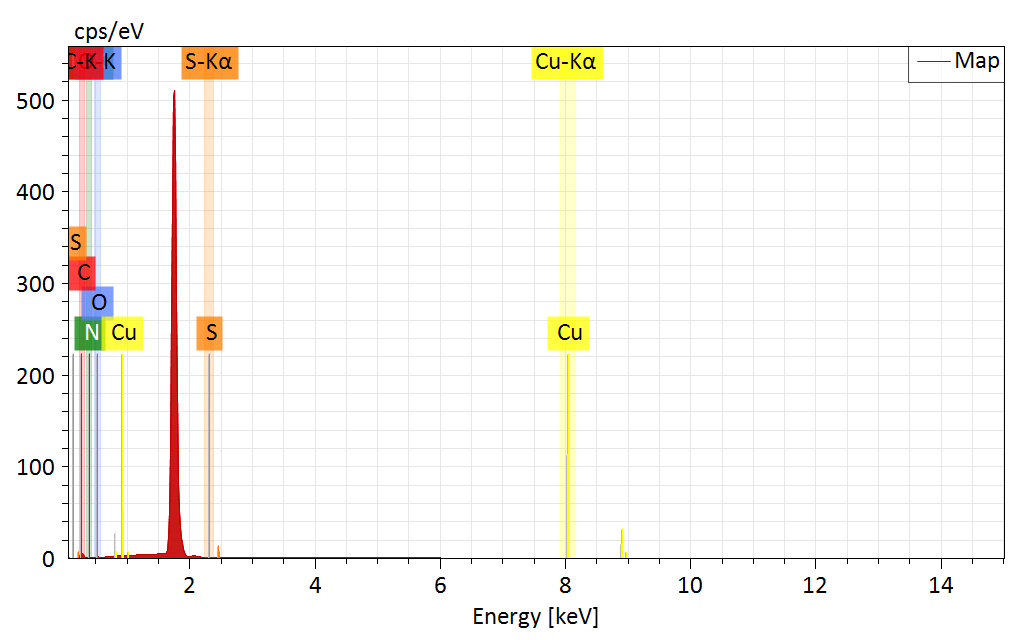


**Figure S6.** Energy-dispersive X-ray spectroscopy analysis of MN NPs.


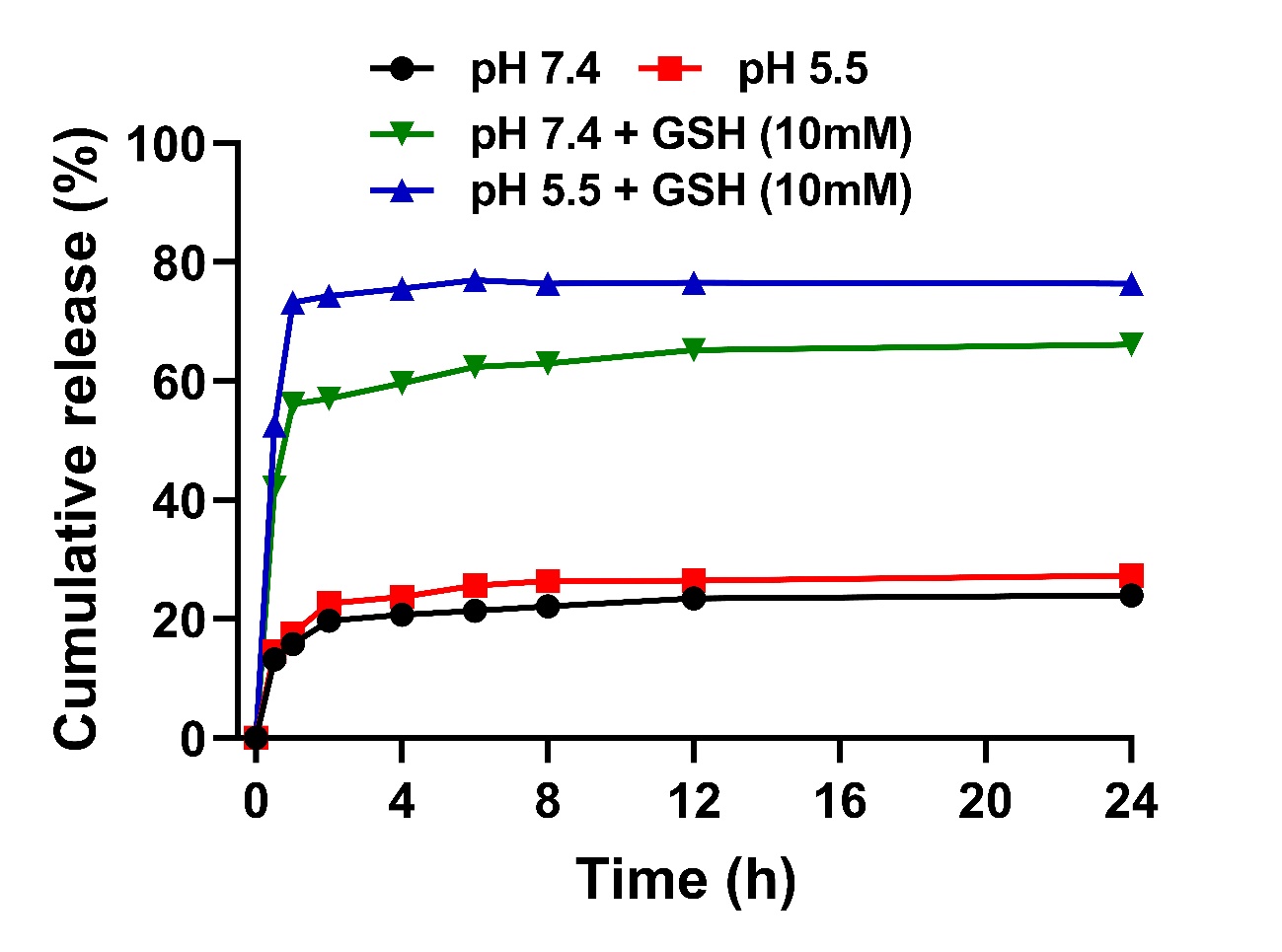


**Figure S7.** Drug-releasing profile of MN NPs in the absence or presence of glutathione (GSH) at varying pH values.


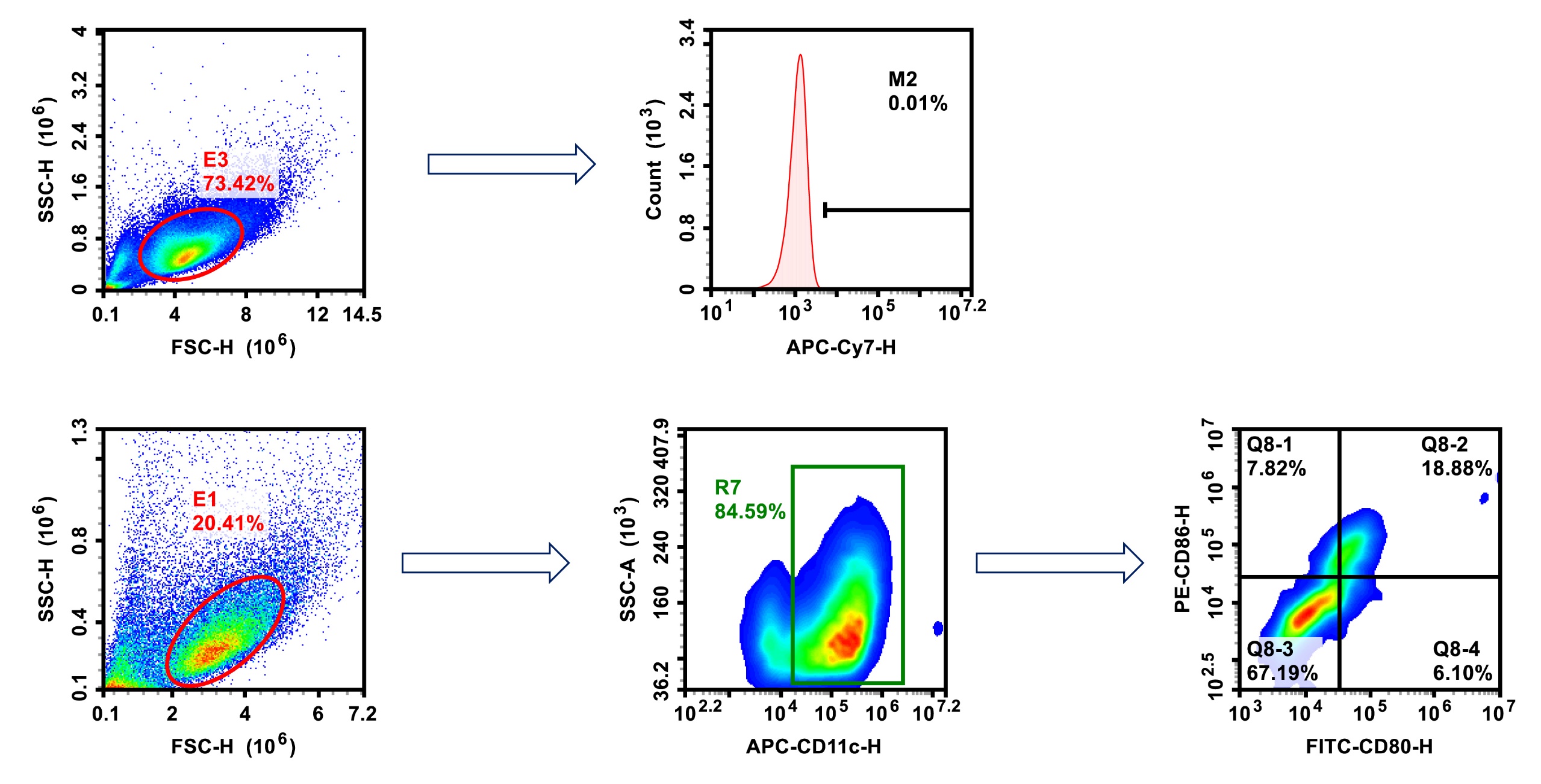


**Figure S8.** Gating strategy for cellular uptake of MN NPs after 24 h incubation. Living cells were identified based on FSC-H versus SSC-H profiles.


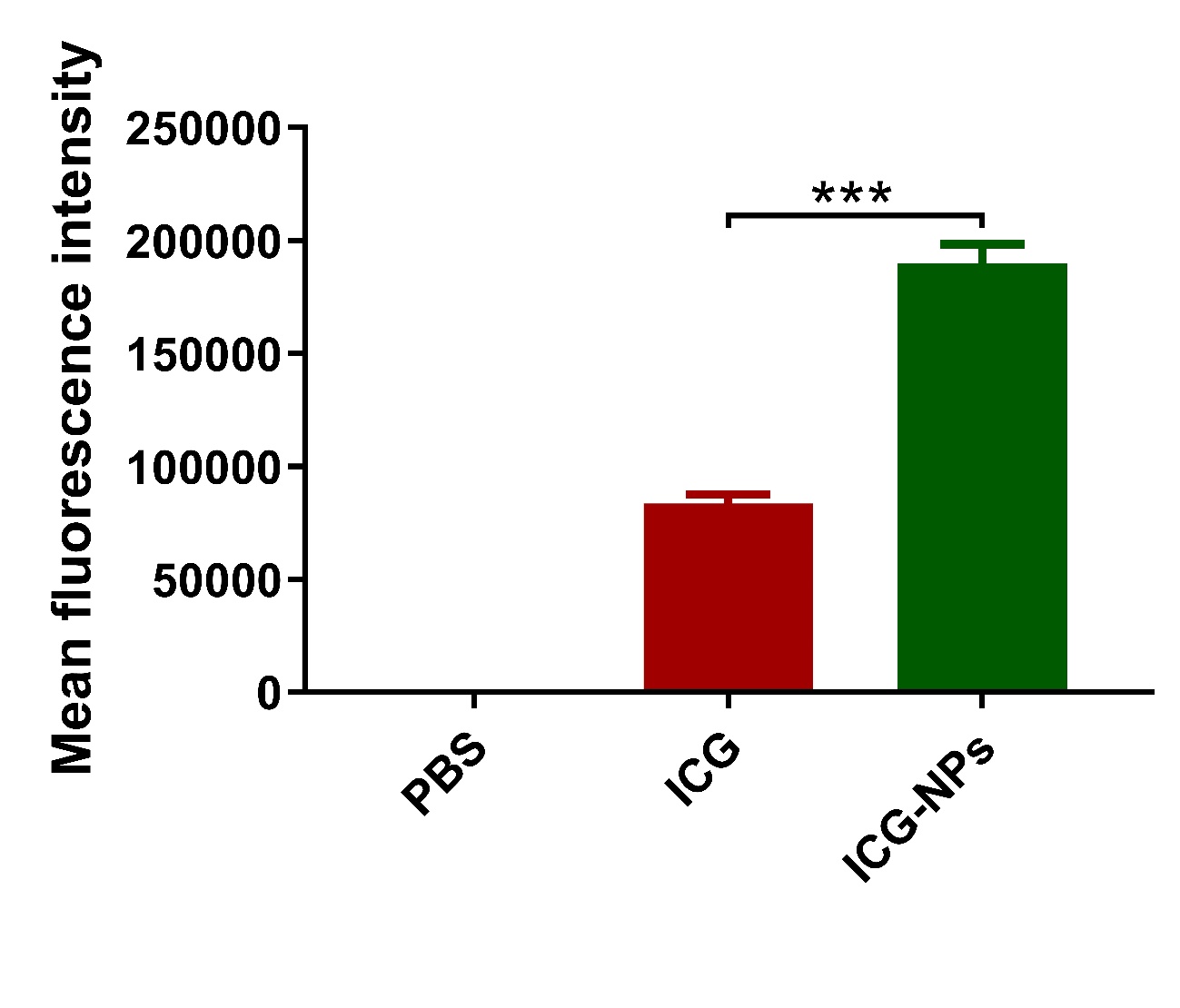


**Figure S9.** Cell uptake after incubation with ICG or ICG-NPs for 24 h. Data are represented as the mean ± SD (n=3). ^***^*P* < 0.001.


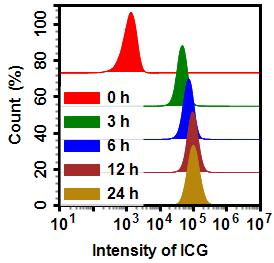

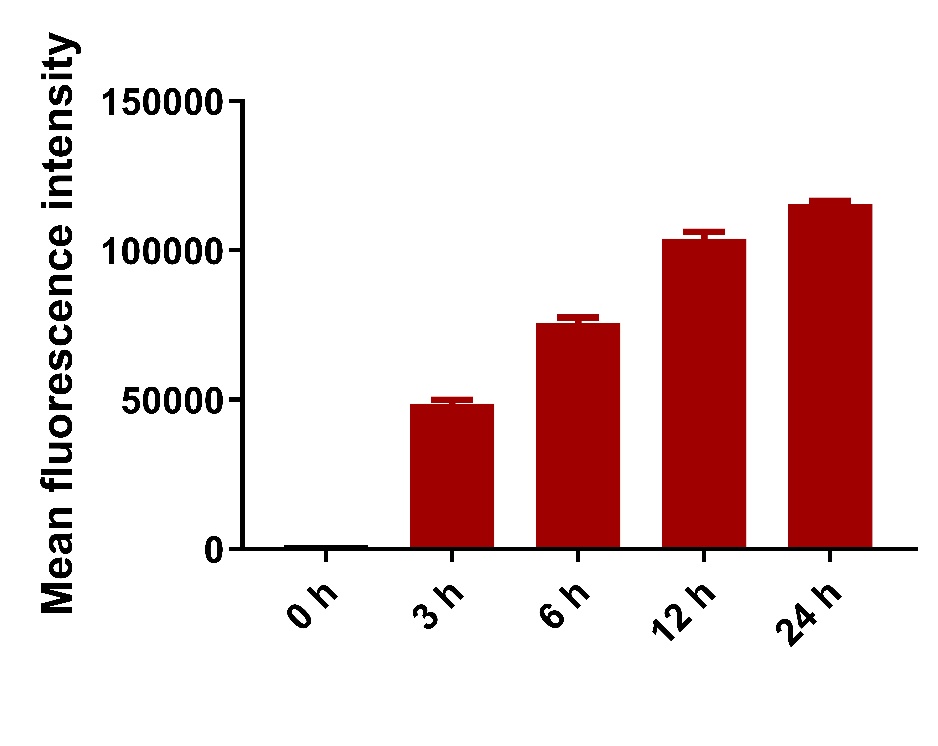


**Figure S10.** Flow cytometry analysis of the cell uptake after incubation with ICG-NPs for different time.


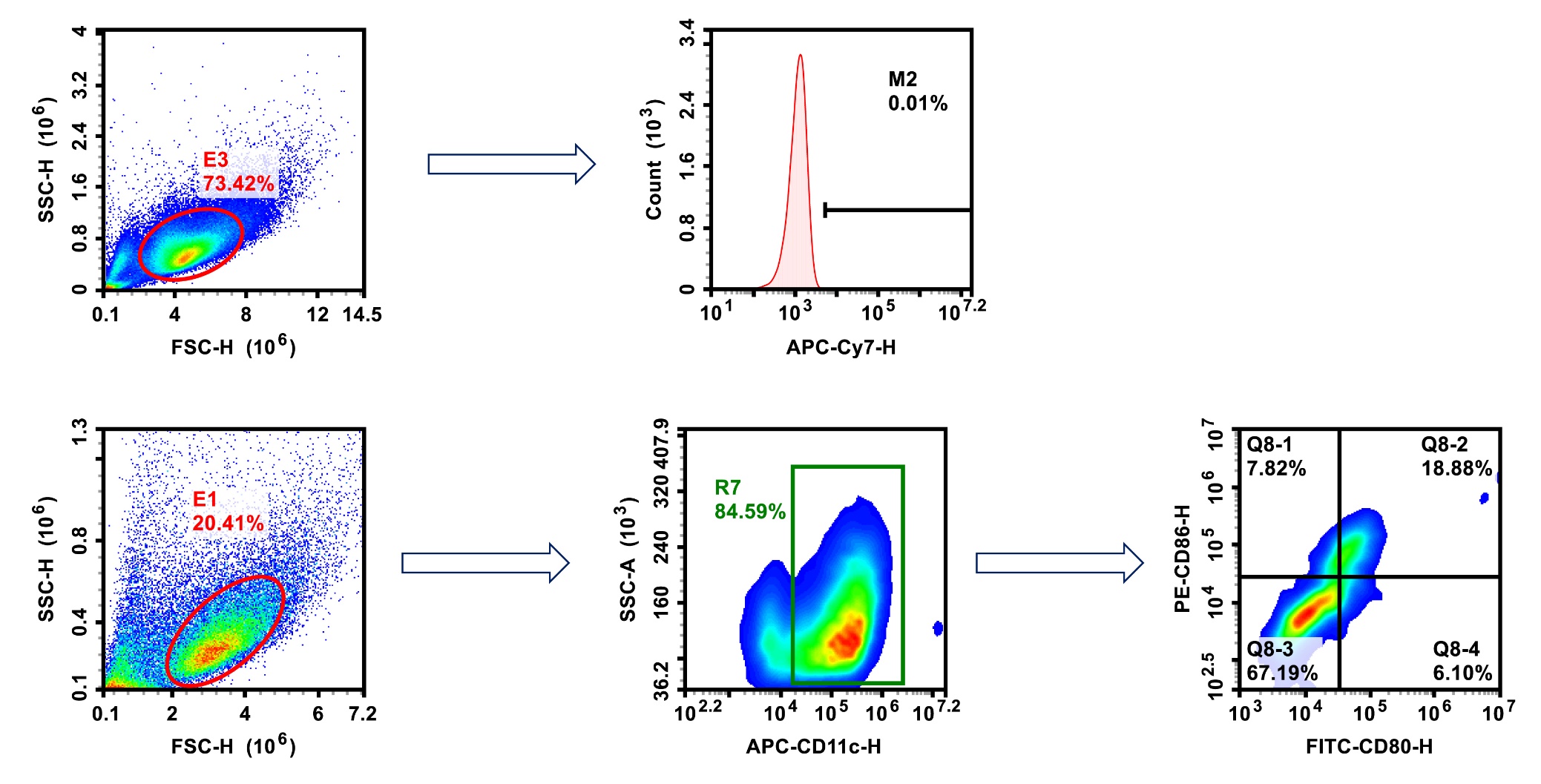


**Figure S11.** Gating strategy for DC maturation in BMDCs or LNs harvested from the immunized mice. Living cells were identified based on FSC-H versus SSC-H profiles. DCs were identified as CD11c^+^.


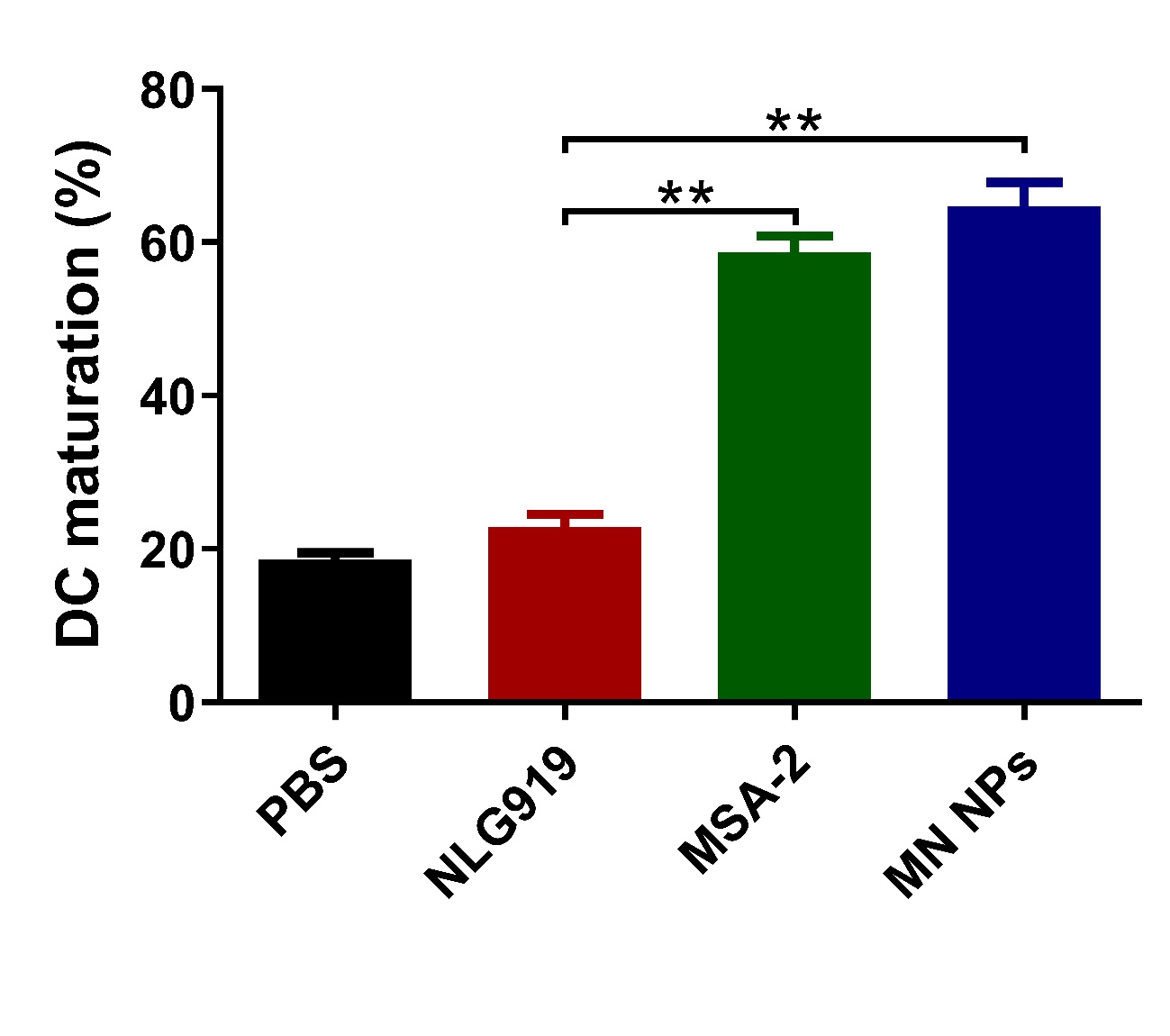


**Figure S12.** Co-expression of CD86 and CD80 on BMDCs. Data are represented as the mean ± SD (n=3). ^*^*P* < 0.05, and ^**^*P* < 0.01.


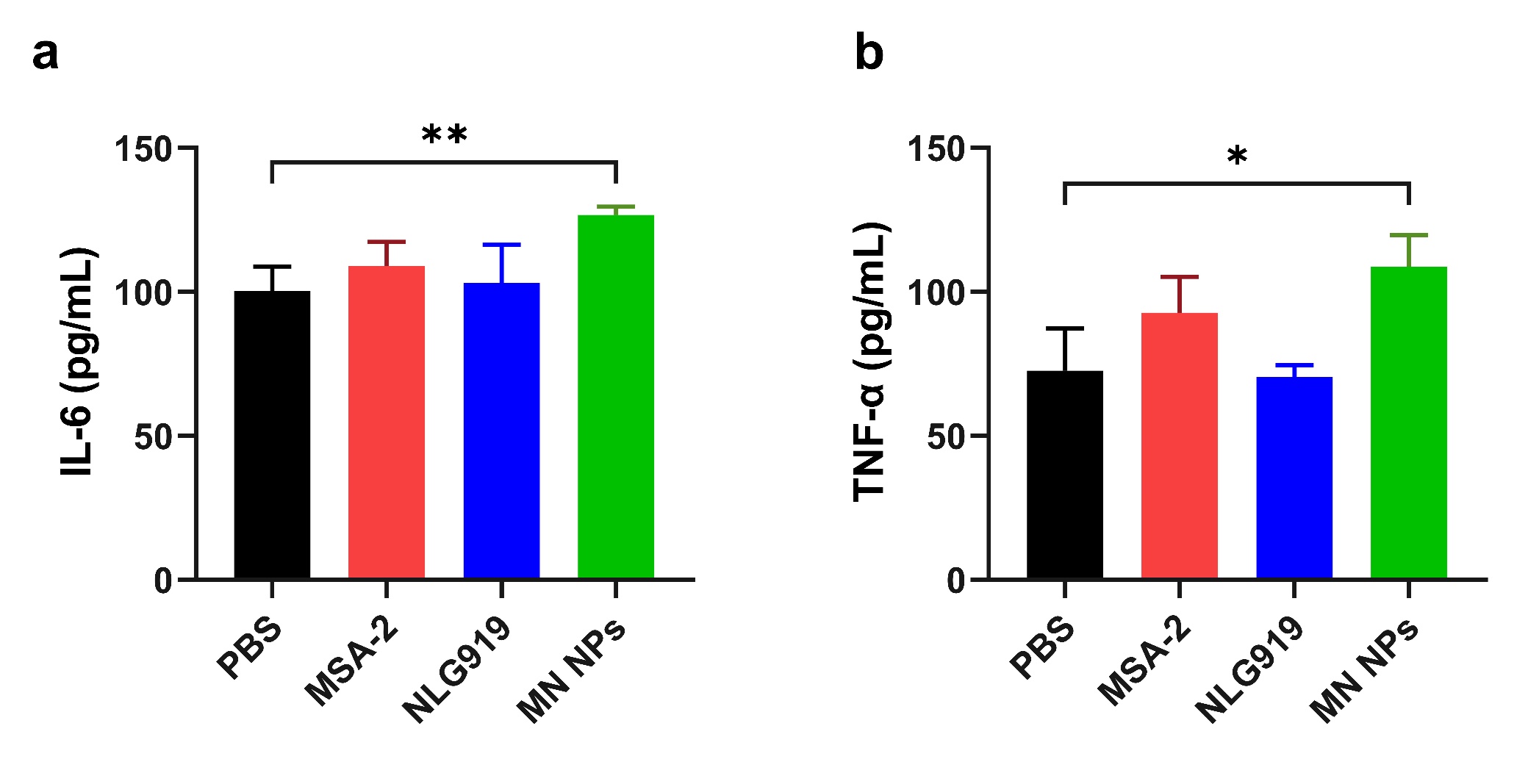


**Figure S13.** IL-6 (a) and TNF-α (b) release levels in BMDCs after various treatments. Data are represented as the mean ± SD (n=5). **P* < 0.05, ***P* < 0.01.


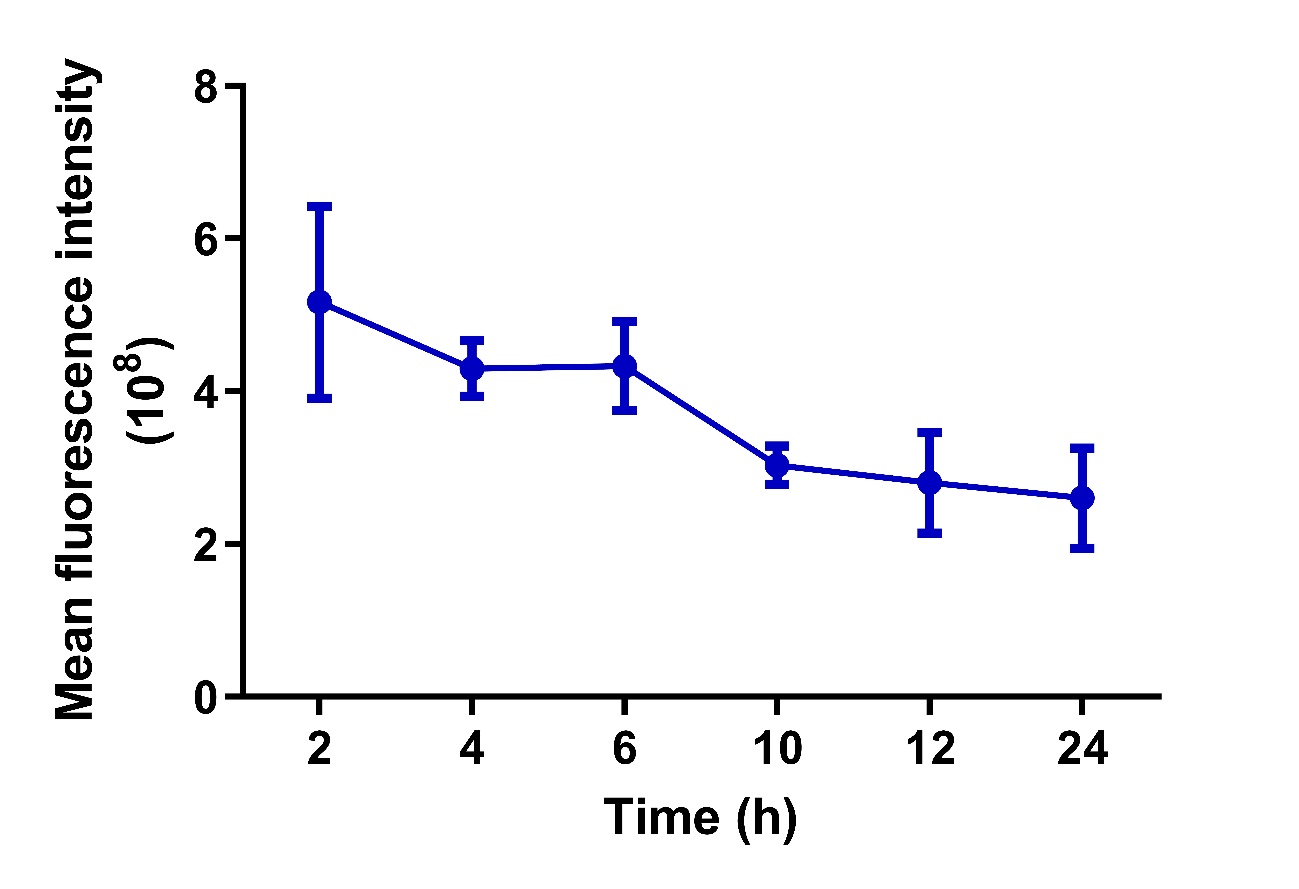


**Figure S14.** Tumor fluorescence intensity at different time points after injection of MN NPs.


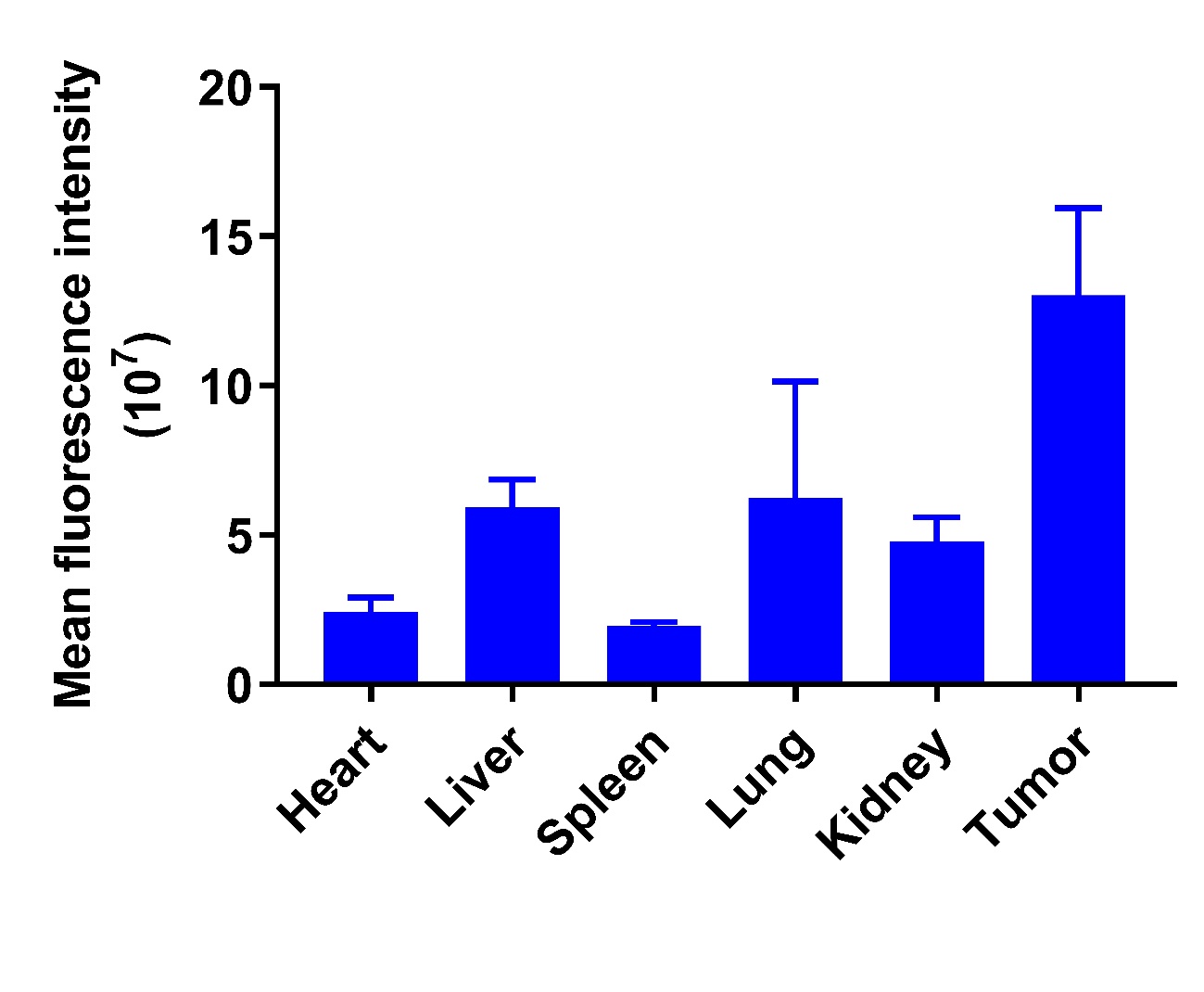


**Figure S15.** Fluorescence intensity of major organs and tumors collected after 24 h post-injection of MN NPs. Data are represented as the mean ± SD (n=3).


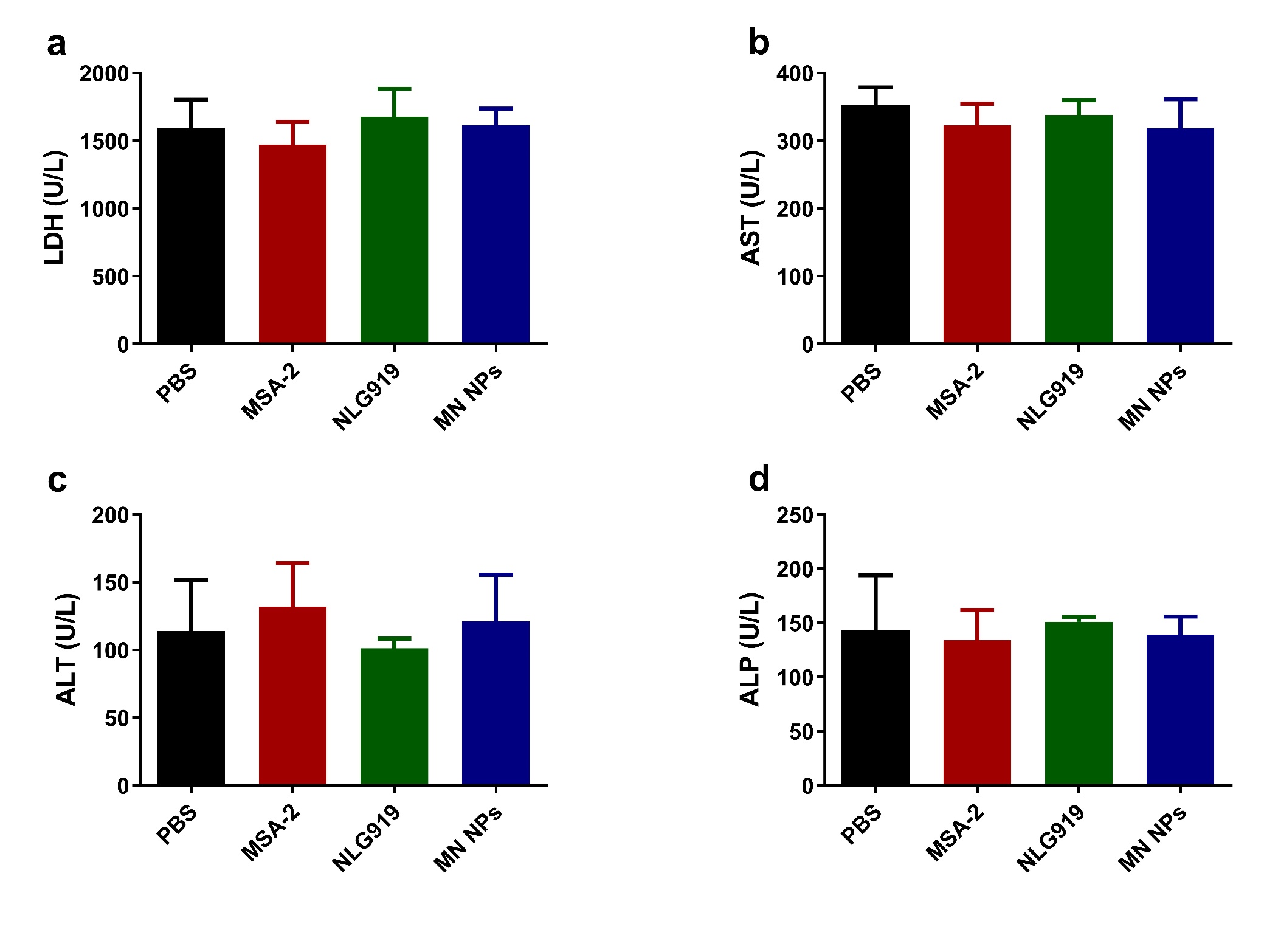


**Figure S16.** The secretion of lactate dehydrogenase (LDH), aspartate transaminase (AST), alanine transaminase (ALT), and alkaline phosphatase (ALP) after different treatments. Data are represented as the mean ± SD (n=6).


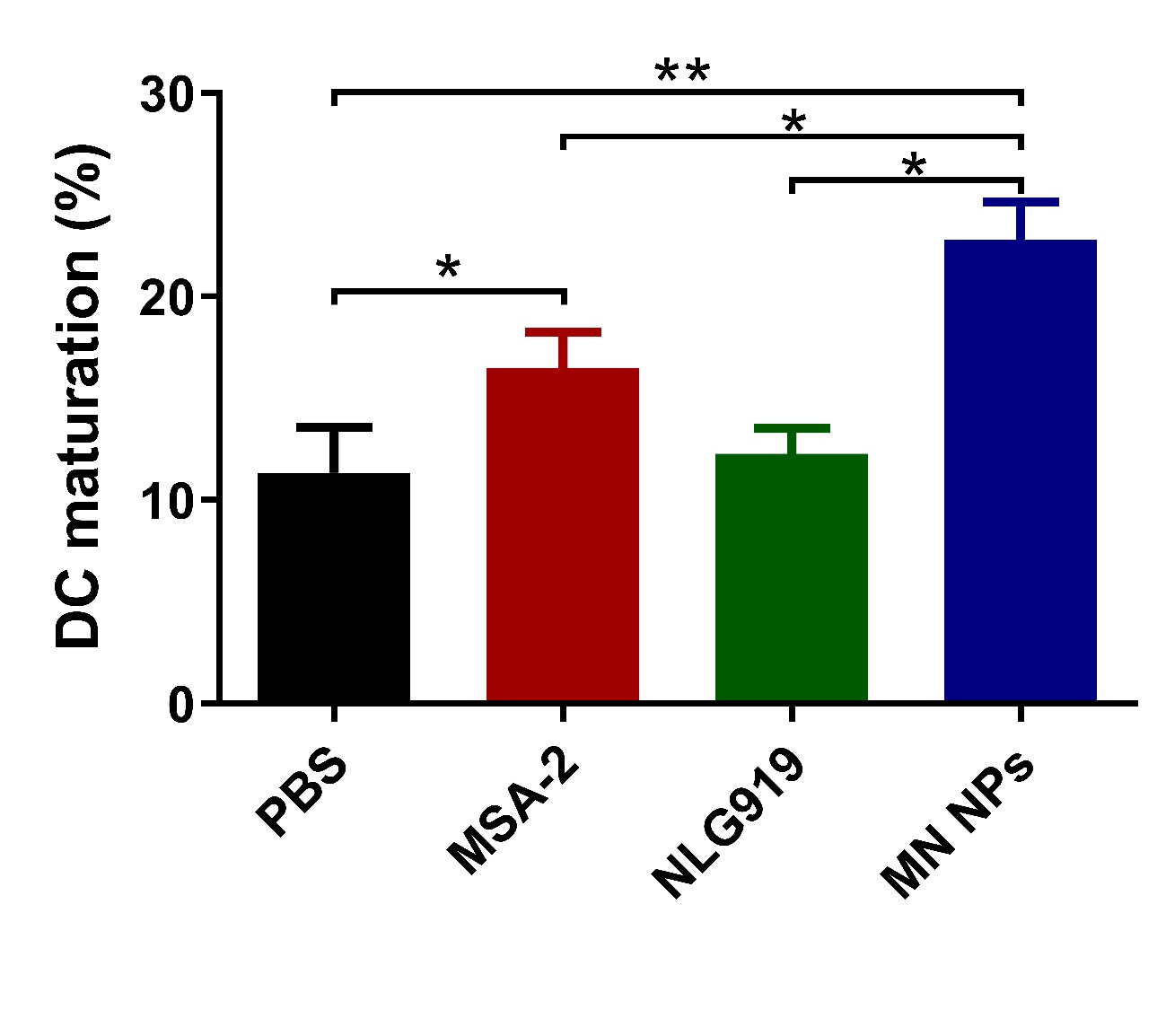


**Figure S17.** Statistical data of DC maturation in the tumor-draining LNs. Data are represented as the mean ± SD (n=6). ^*^*P* < 0.05, and ^**^*P* < 0.01.


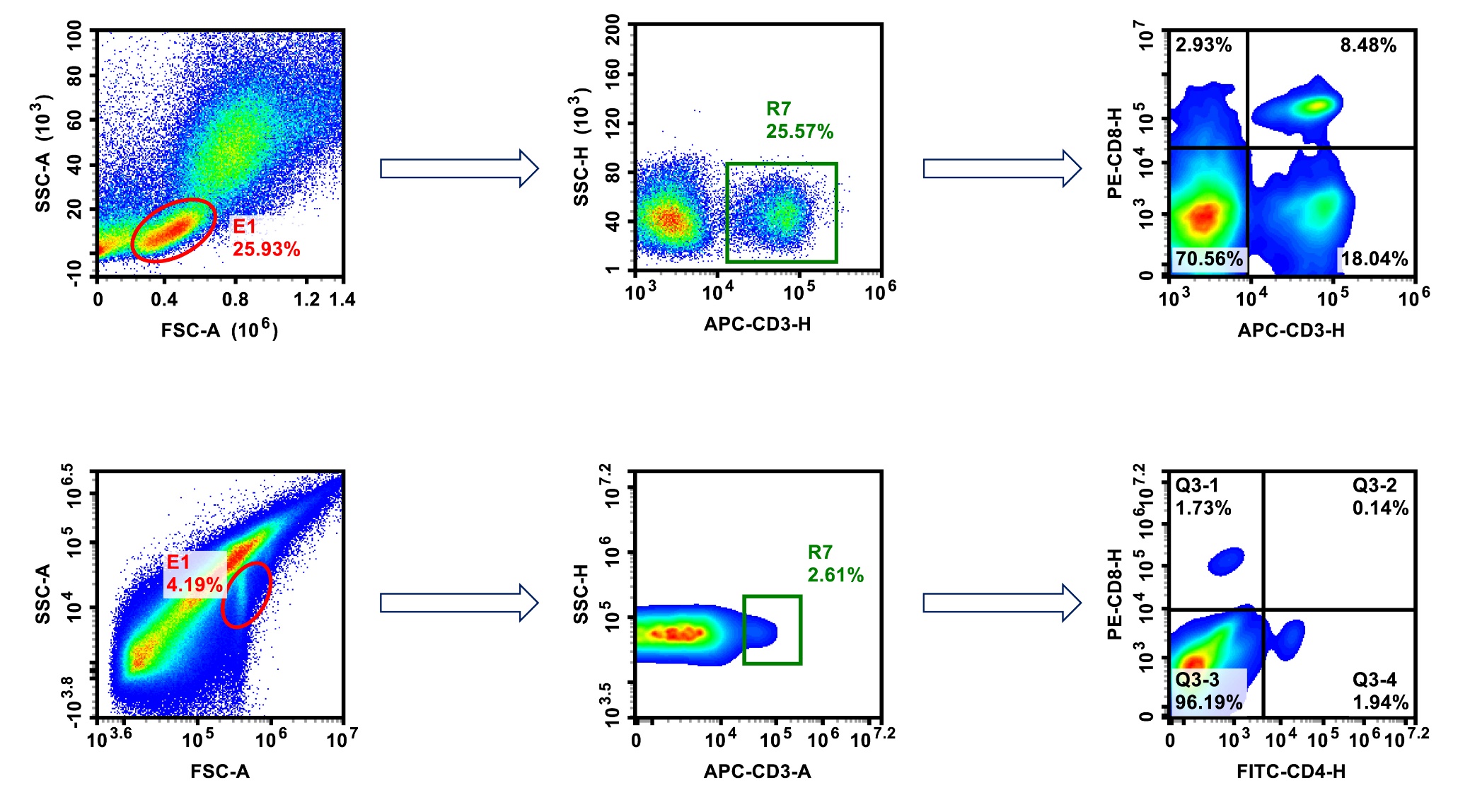


**Figure S18.** Gating strategy for detection of CD3^+^CD8^+^ T cells in spleen harvested from immunized mice. Living cells were identified based on FSC-A versus SSC-A profiles.


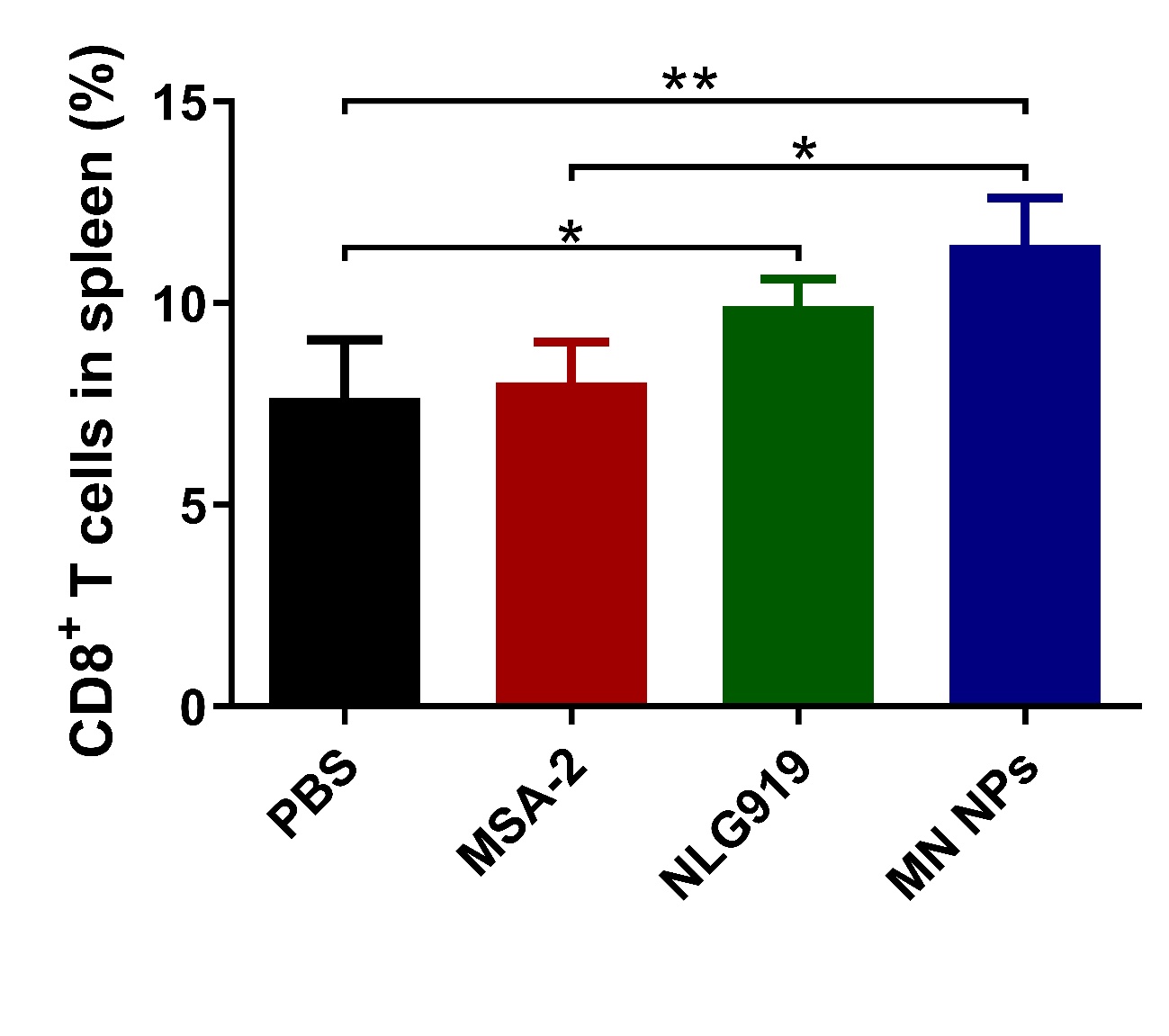


**Figure S19.** CD3^+^CD8^+^ T cells in spleen harvested from the mice treated with different formulations. Data are represented as the mean ± SD (n=6). ^*^*P* < 0.05, ^**^*P* < 0.01.


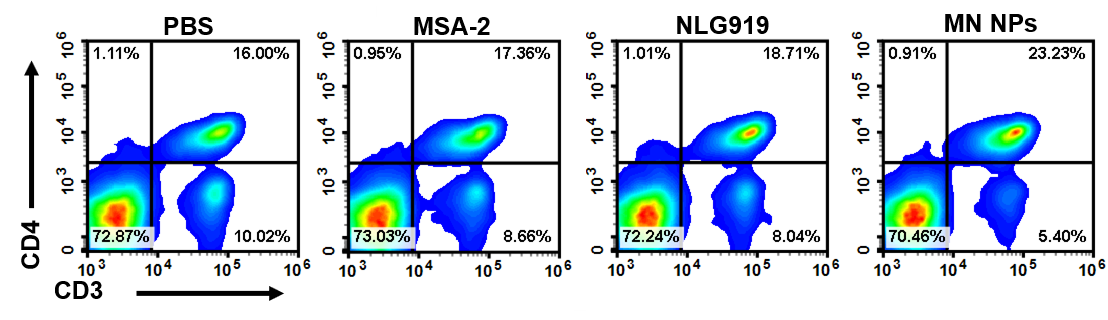


**Figure S20.** Representative flow cytometry analysis of CD3^+^CD4^+^ T cells in spleen harvested from the mice treated with different formulations.


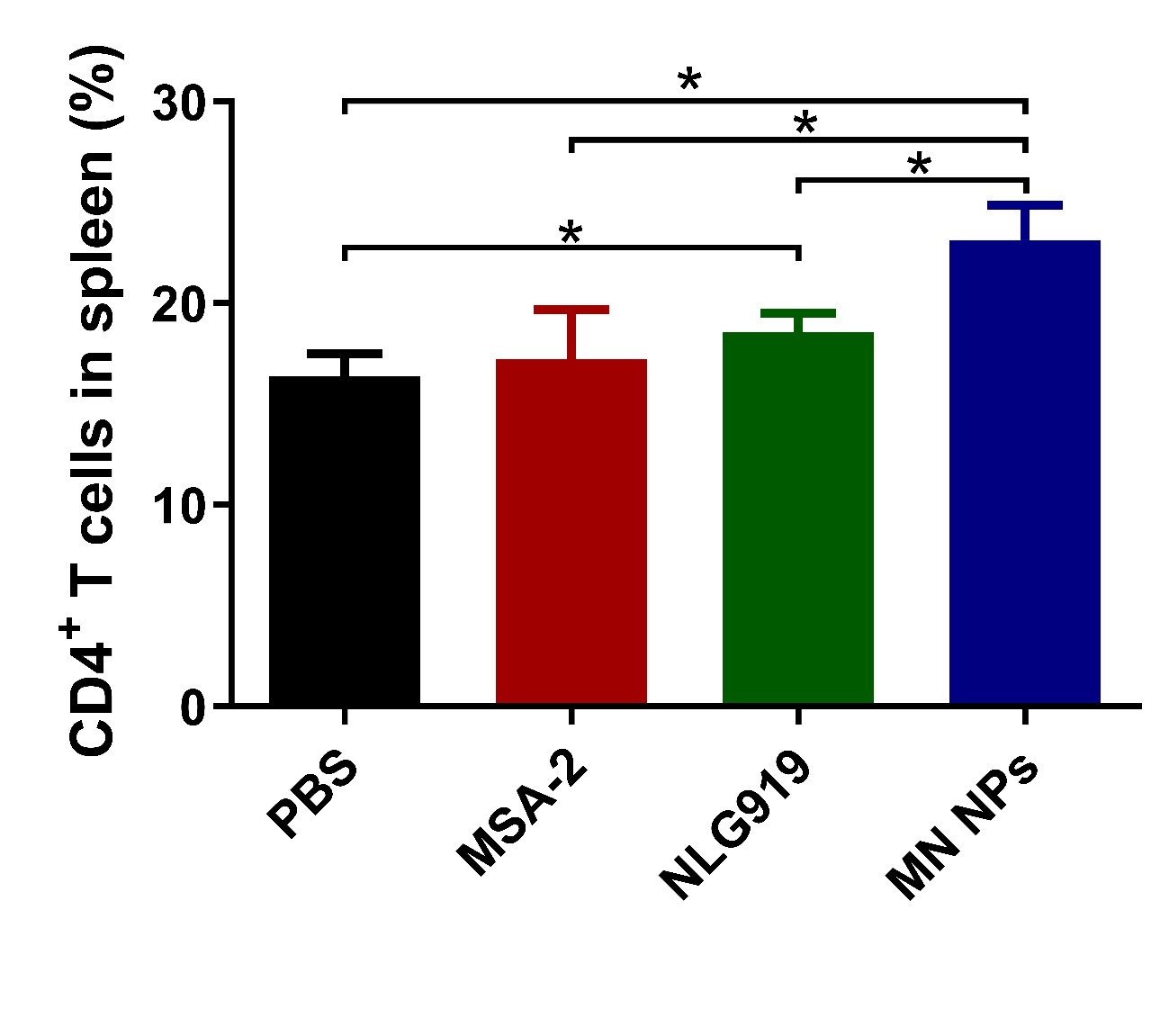


**Figure S21.** CD3^+^CD4^+^ T cells in spleen harvested from the mice treated with different formulations. Data are represented as the mean ± SD (n=6). ^*^*P* < 0.05, ^**^*P* < 0.01.


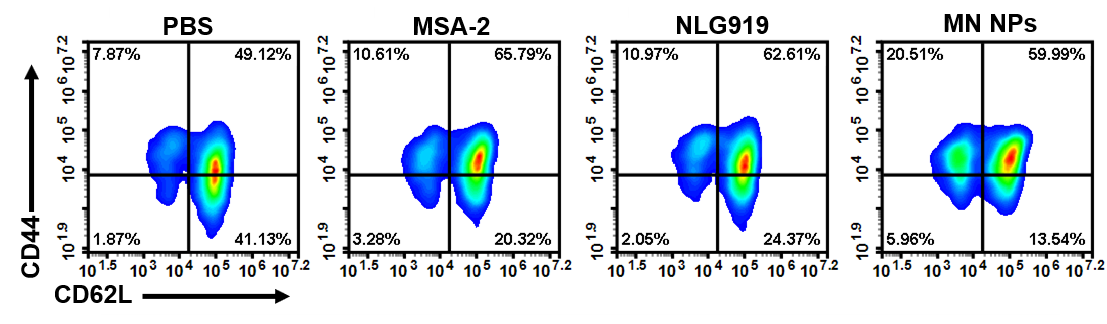


**Figure S22.** Representative flow cytometry analysis of effector memory T cells (T_EM_, CD8^+^CD44^+^CD62L^-^) in spleen harvested from the mice treated with different formulations.


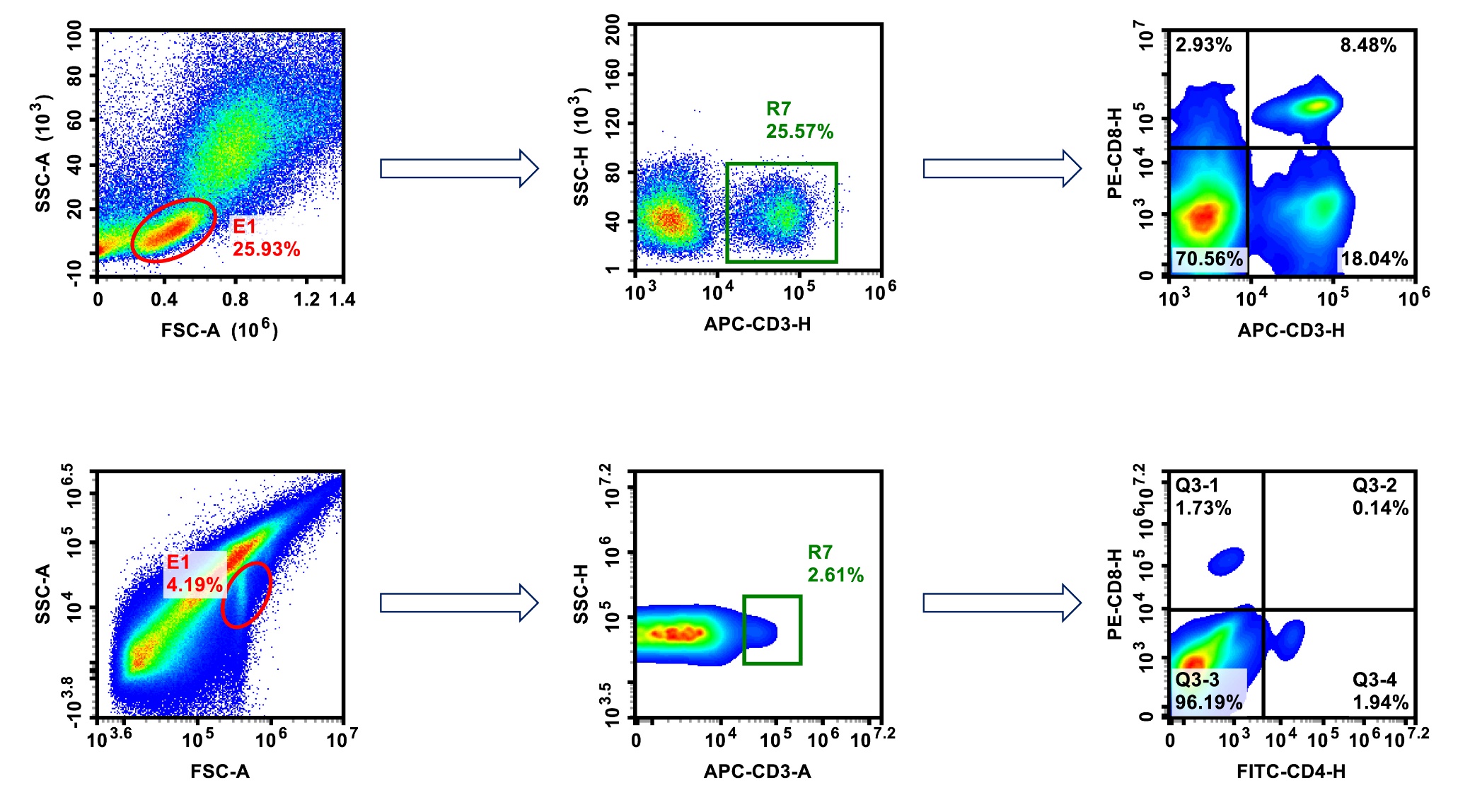


**Figure S23.** Gating strategy for detection of CD3^+^CD4^+^ and CD3^+^CD8^+^ T cells in tumors harvested from immunized mice. Living cells were identified based on FSC-A versus SSC-A profiles.


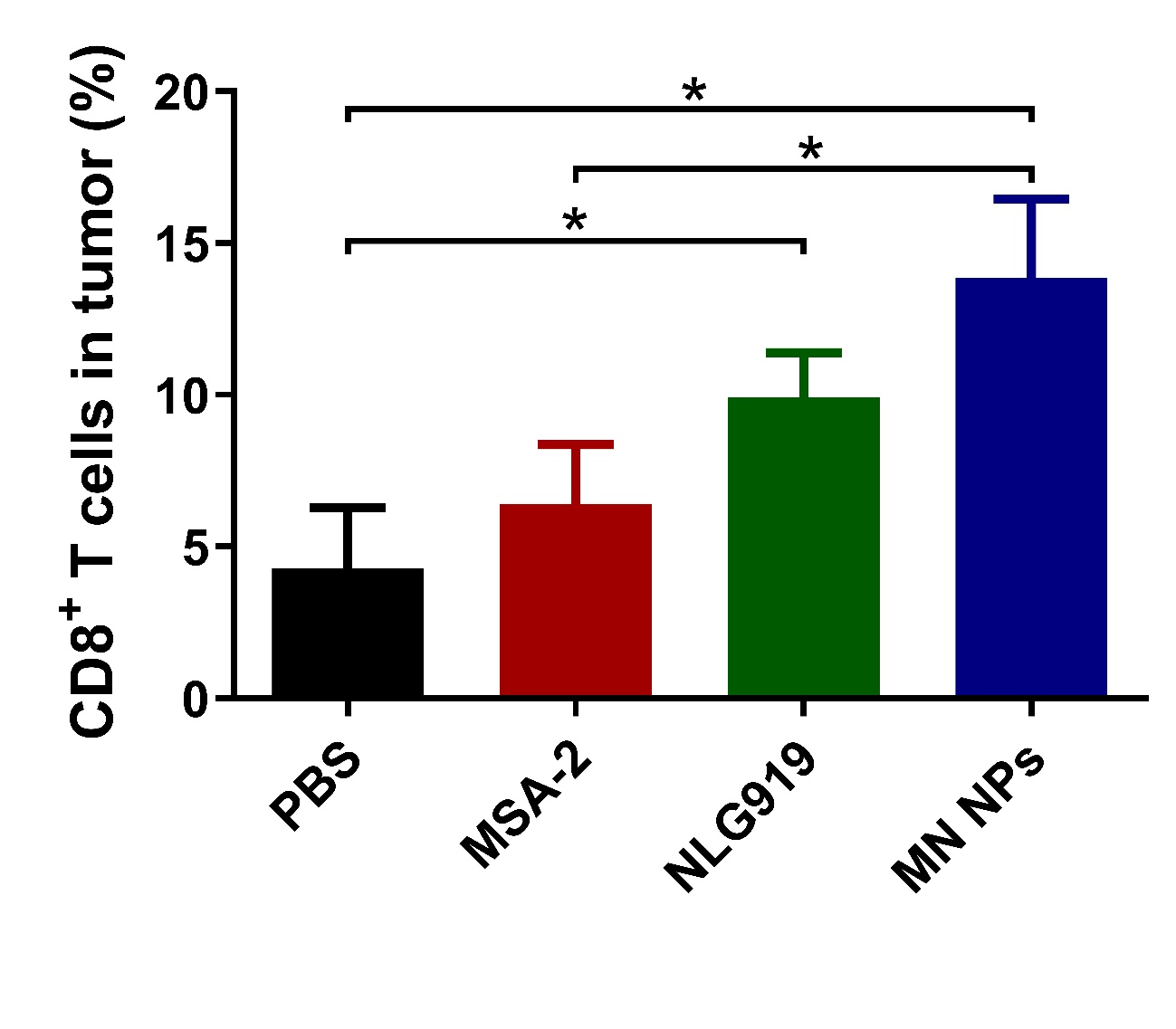


**Figure S24.** CD3^+^CD8^+^ T cells in tumors harvested from the mice treated with different formulations. Data are represented as the mean ± SD (n=6). ^*^*P* < 0.05, ^**^*P* < 0.01.


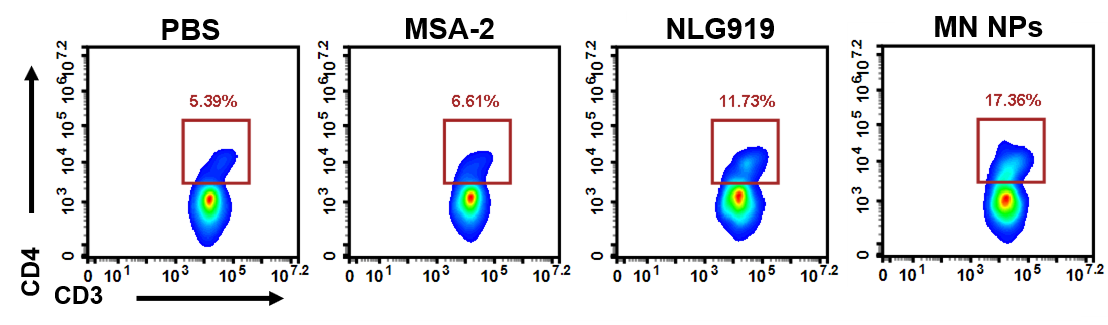


**Figure S25.** Representative flow cytometry analysis of CD3^+^CD4^+^ T cells in tumors harvested from the mice treated with different formulations.

**
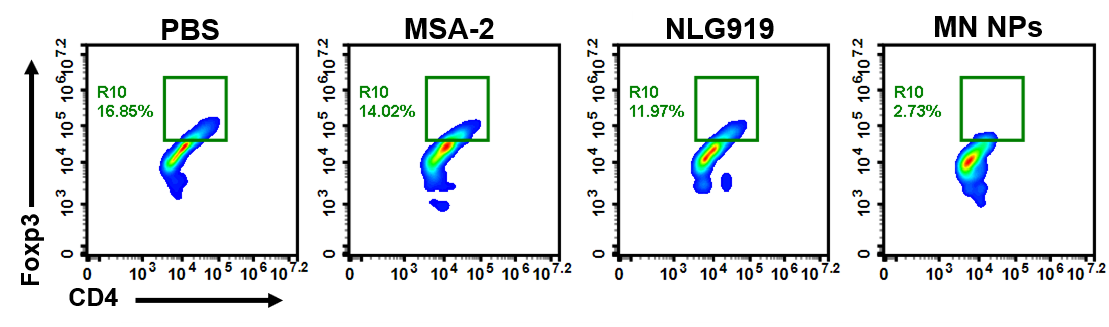
**

**Figure S26.** Representative flow cytometry analysis of regulatory T cells (Tregs, CD4^+^Foxp3^+^) in tumors harvested from the mice treated with different formulations.

**
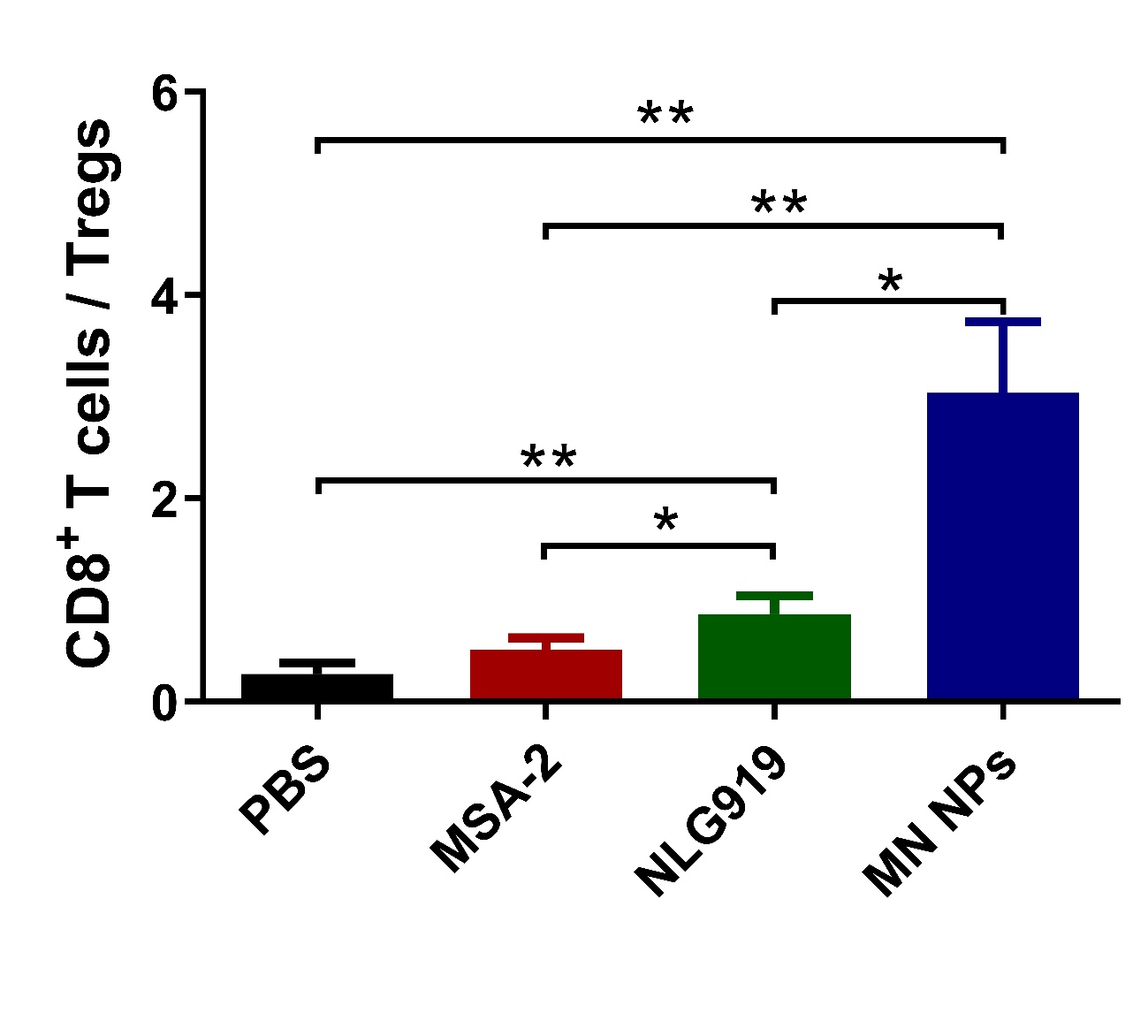
**

**Figure S27.** The ratio of CD8^+^ T cells and Tregs in tumors. Data are represented as the mean ± SD (n=6). ^*^*P* < 0.05, ^**^*P* < 0.01.


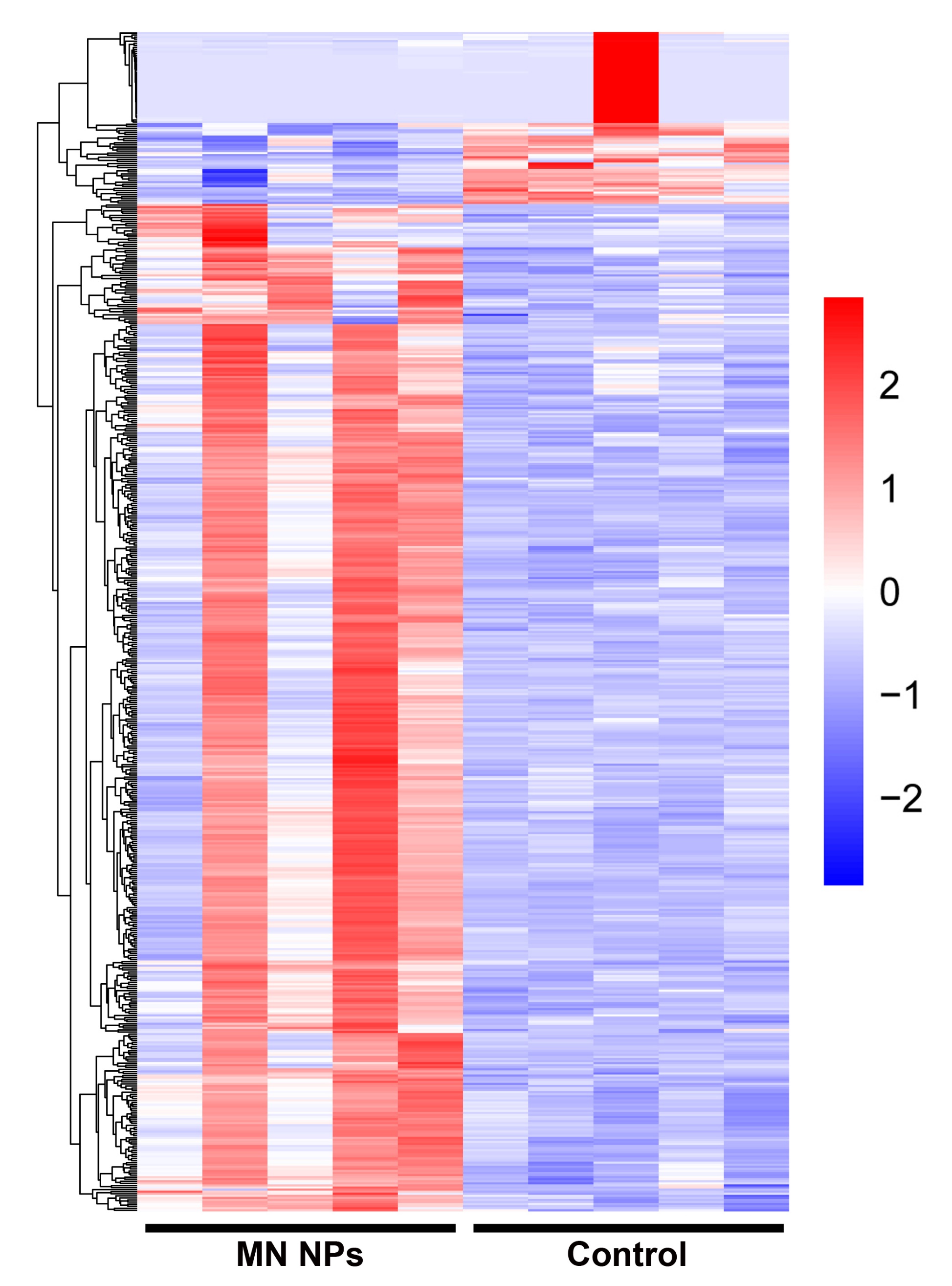


**Figure S28.** Heat map of differentially expressed genes in tumors after the treatment. Red and blue color represent upregulation and downregulation, respectively (n=5).


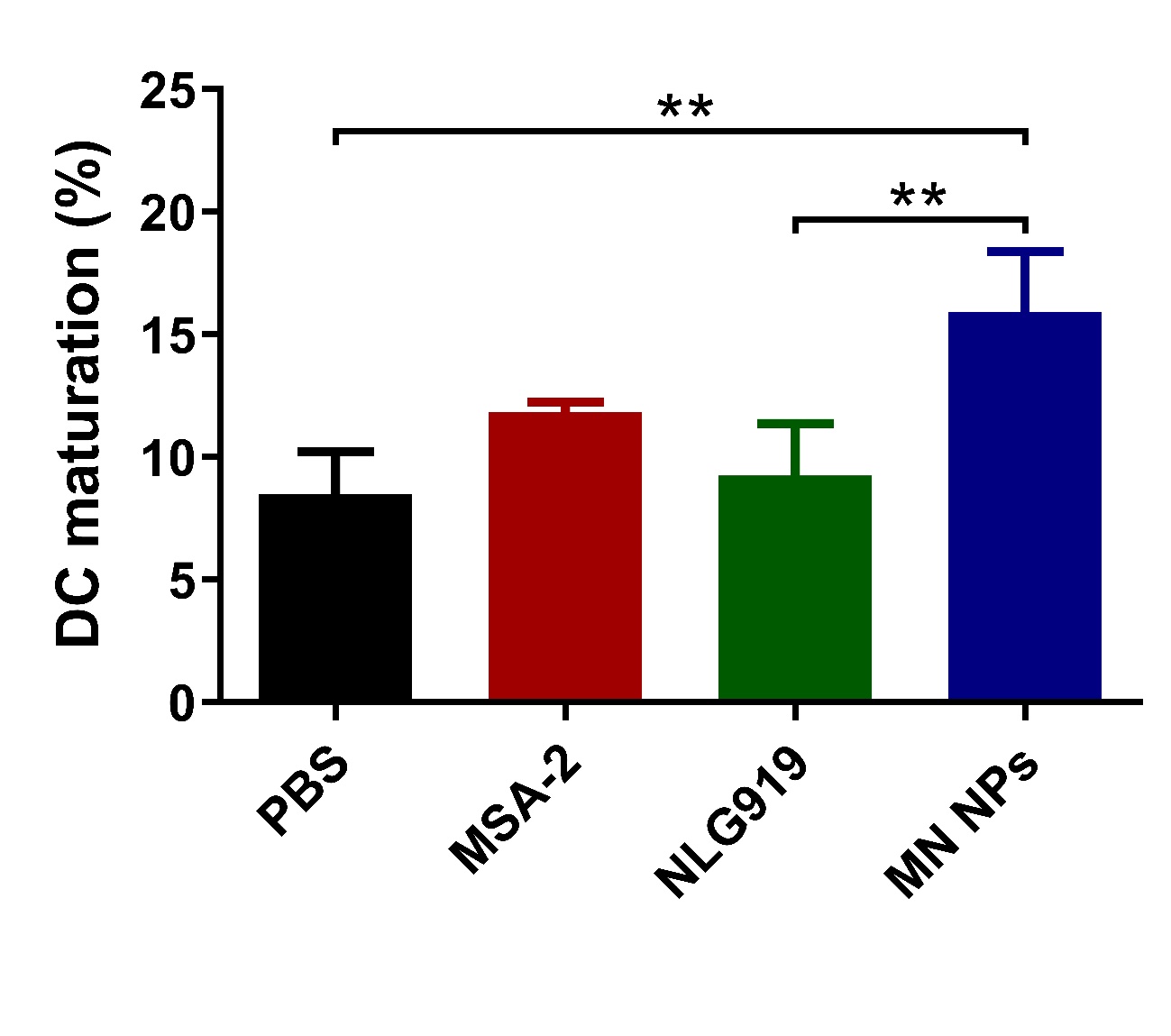


**Figure S29.** Statistical data of DC maturation in the tumor-draining LNs of mice bearing B16 tumors. Data are represented as the mean ± SD (n=6). ^**^*P* < 0.01.


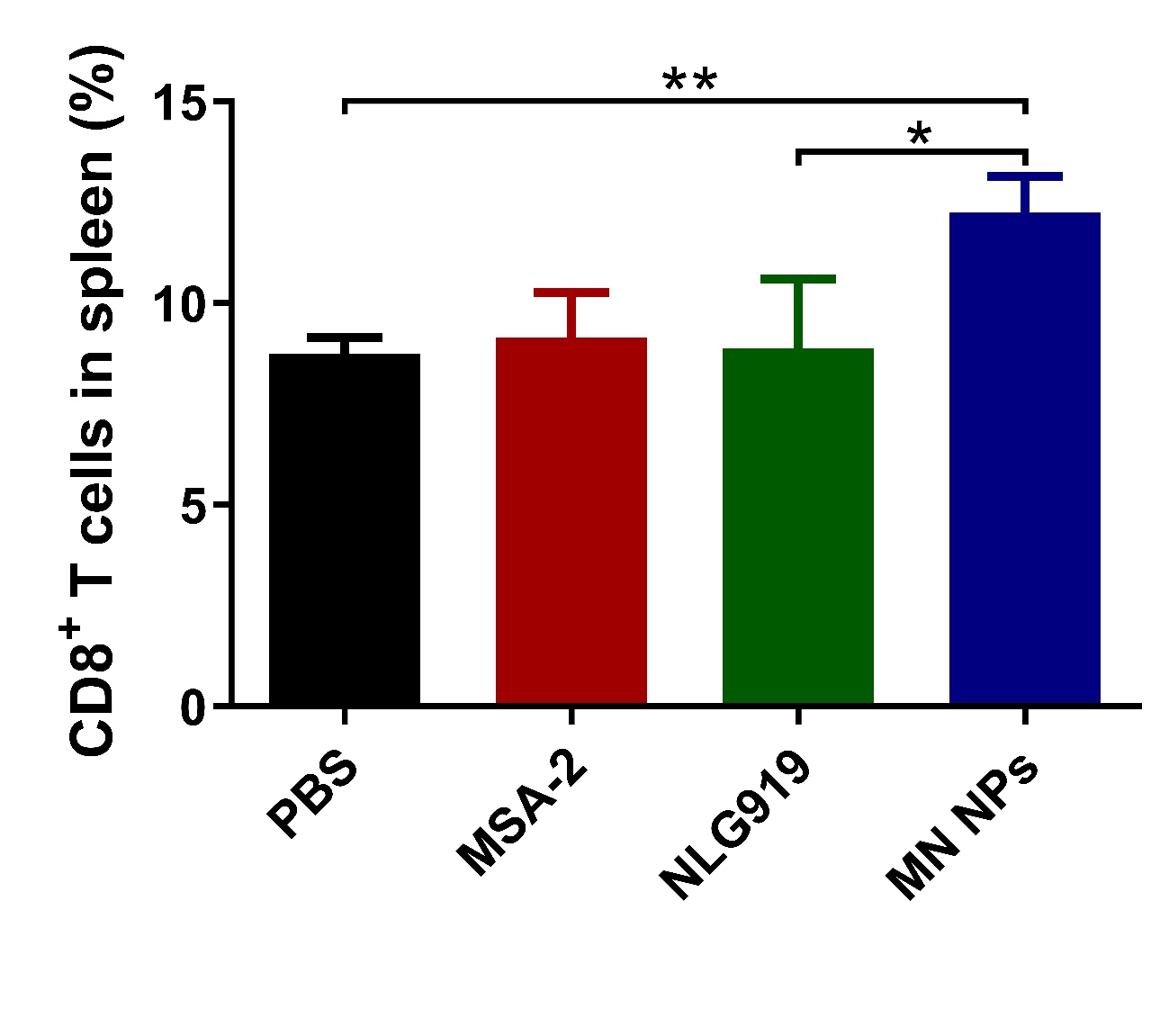


**Figure S30.** CD3^+^CD8^+^ T cells in spleen harvested from the mice bearing B16 tumors treated with different formulations. Data are represented as the mean ± SD (n=6). ^*^*P* < 0.05, ^**^*P* < 0.01.


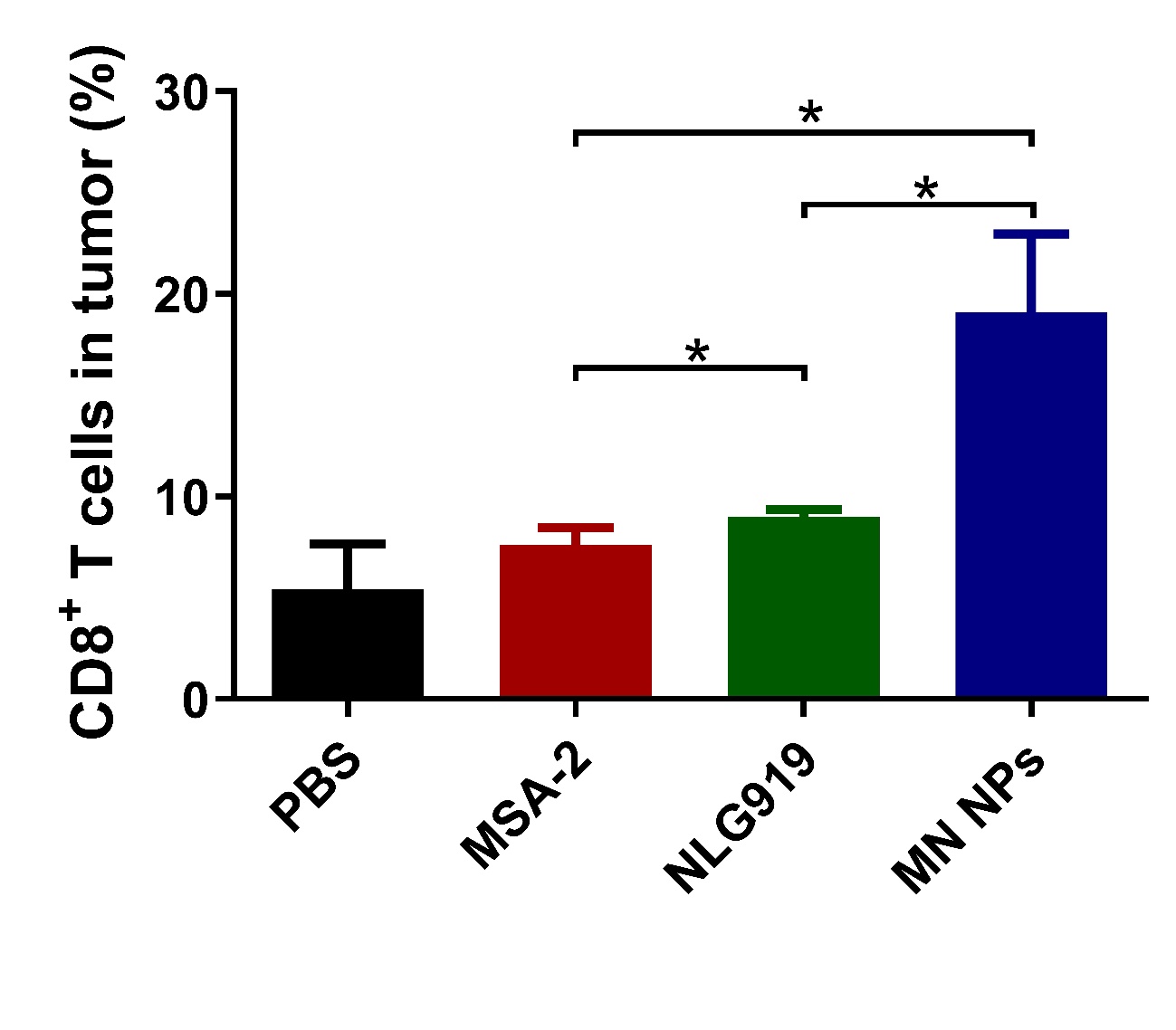


**Figure S31.** CD3^+^CD8^+^ T cells in tumors harvested from the mice bearing B16 tumors treated with different formulations. Data are represented as the mean ± SD (n=6). ^*^*P* < 0.05.


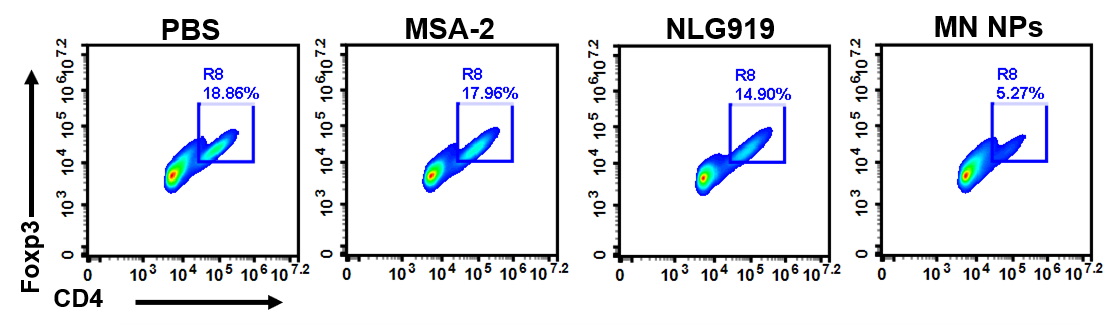


**Figure S32.** Representative flow cytometry analysis of regulatory T cells (Tregs, CD4^+^Foxp3^+^) in tumors harvested from the mice bearing B16 tumors treated with different formulations.


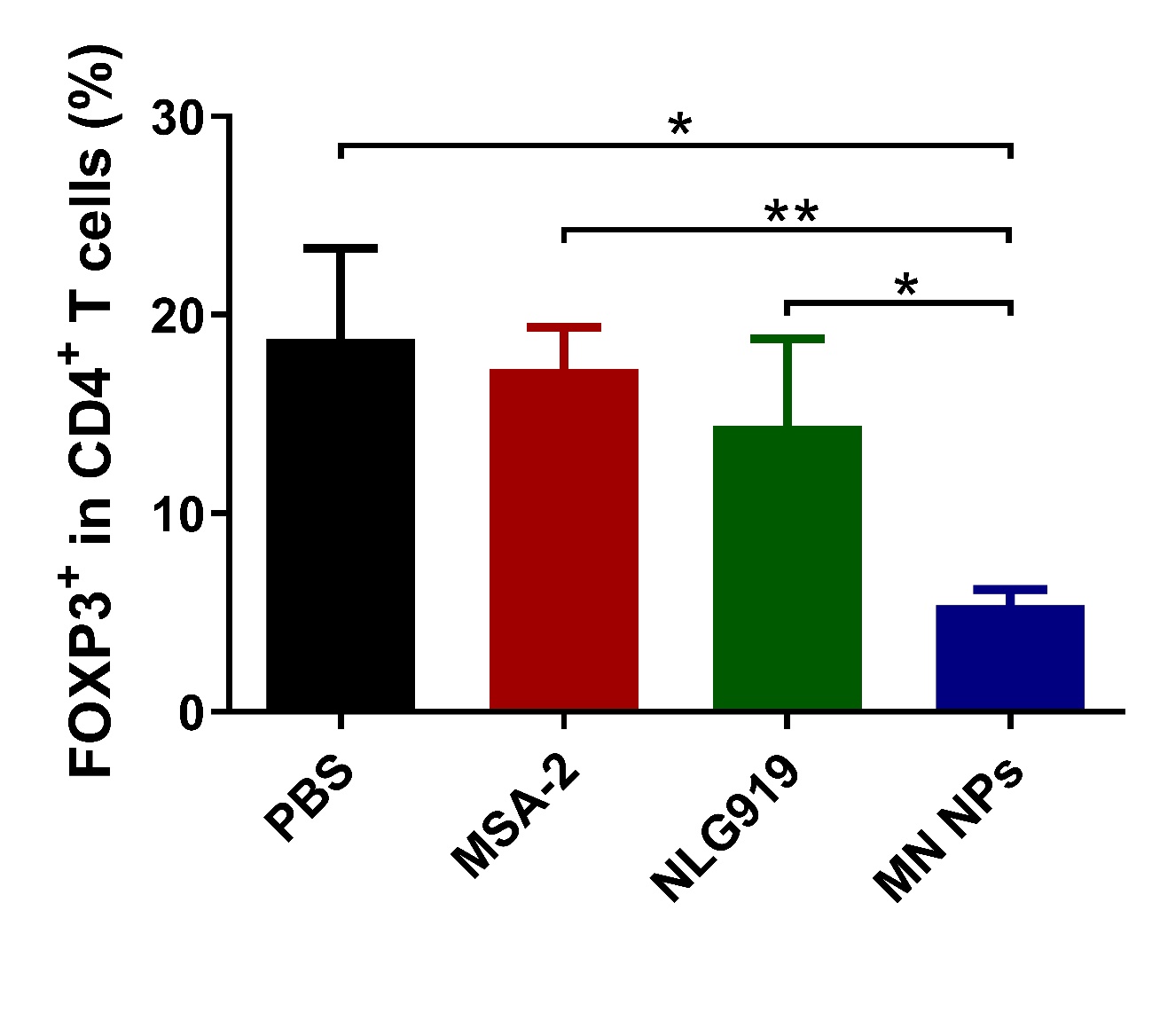


**Figure S33.** Tregs (CD4^+^Foxp3^+^) in tumors harvested from the mice bearing B16 tumors treated with different formulations. Data are represented as the mean ± SD (n=6). ^*^*P* < 0.05, ^**^*P* < 0.01.


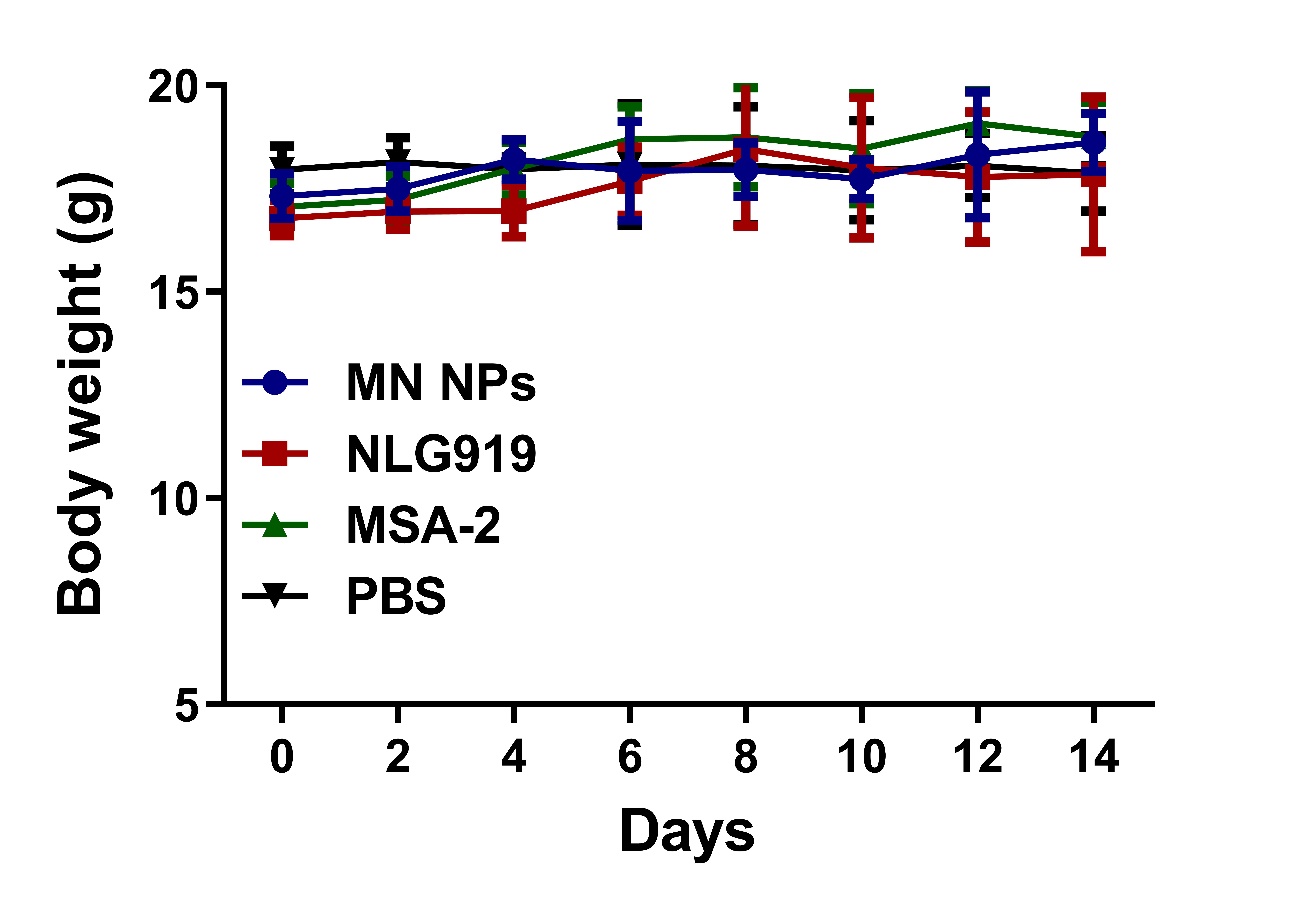


**Figure S34.** Body weight changes of mice bearing B16 tumors after different treatments. Data are represented as the means ± SD (n=6).
